# Dissolution-redeposition dynamics as a universal restructuring mechanism of electrocatalysts

**DOI:** 10.1016/j.checir.2026.100049

**Published:** 2026-09-01

**Authors:** Blaž Tomc, Milutin Smiljanić, Mitja Kostelec, Mejrema Nuhanović, Muhammad Zahid, Leonard Moriau, Ana Rebeka Kamšek, Aleš Marsel, Miha Hotko, Nik Maselj, Ante Matošin, Ana Herceg, Lazar Bijelić, Ožbej Vodeb, Anja Logar, Madis Lüsi, Jan Ocepek, Angelja Kjara Surca, Črtomir Donik, Irena Paulin, Matjaž Godec, Luka Suhadolnik, Francisco Ruiz-Zepeda, Primož Jovanovič, Martin Šala, Marjan Bele, Nejc Hodnik

**Affiliations:** 1 National Institute of Chemistry, 1000 Ljubljana, Slovenia; 2 University of Nova Gorica, 5000 Nova Gorica, Slovenia; 3 Institute of Metals and Technology, 1000 Ljubljana, Slovenia; 4 Jozef Stefan International Postgraduate School, 1000 Ljubljana, Slovenia

**Keywords:** electrocatalysis, metals, dissolution, redeposition, Ostwald ripening, transient dissolution, structure-property relationship, fuel cell, electrolysis, recycling

## Abstract

Electrocatalysts are often assumed to be structurally stable; yet in practice, they continuously dissolve and restructure, directly affecting the number and nature of active sites and therefore performance. Although dissolution-redeposition-driven evolution is well recognized in some electrocatalysis communities, others still interpret activity and selectivity as properties of static surfaces. We argue that across electrocatalytic technologies, especially under demanding industrially relevant timescales, “static” electrocatalysts are a myth: even parts-per-billion metal fluxes can cumulatively change surface structure, poison membranes, degrade supports, and thus shift device performance. Controlling these changes requires a mechanistic understanding of when dissolution, transport, and redeposition are promoted or suppressed by, for instance, potential, pH, reactants, or local microenvironments. Here, we unify evidence for restructuring across metal and metal-derived catalysts under various electrochemical reactions, highlight common driving forces, and summarize the appropriate tools to quantify it. Ultimately, electrocatalysts should be evaluated, designed, and interpreted as dynamically stable materials and not as static electrochemical interfaces.

## Context & scale

The chemicals and fuels industry is electrifying to meet climate goals, shifting production away from fossil feedstocks and toward renewable electricity for green hydrogen, ammonia, and other energy-intensive commodities. This transition rests on electrolyzers and fuel cells and thus on electrocatalysts that are not only efficient but also durable over industrial lifetimes. Yet most leading candidates are metals or metal-derived materials, which are intrinsically dynamic under realistic operating conditions: atoms dissolve, migrate, and redeposit; surfaces restructure; and active sites evolve over time. Static structure-activity relationships can therefore fail precisely at the atomic scale, where selectivity and degradation are determined, making performance and stability history dependent rather than fixed. Recognizing atomic-level cycling also reconnects electrocatalysis to corrosion science, hydrometallurgy, and circular materials management, as the same elementary steps govern atom loss, transport, and recovery. Treating atomic-level cycling as a design variable enables durability metrics, stability protocols, and materials choices that either suppress harmful pathways or steer benign self-regeneration. It also opens routes to lower-impact repair, recovery, reuse, and recycling of critical metals, aligning catalyst design with resource efficiency and a truly circular, electrified process industry.

## Graphical abstract

**Figure:**
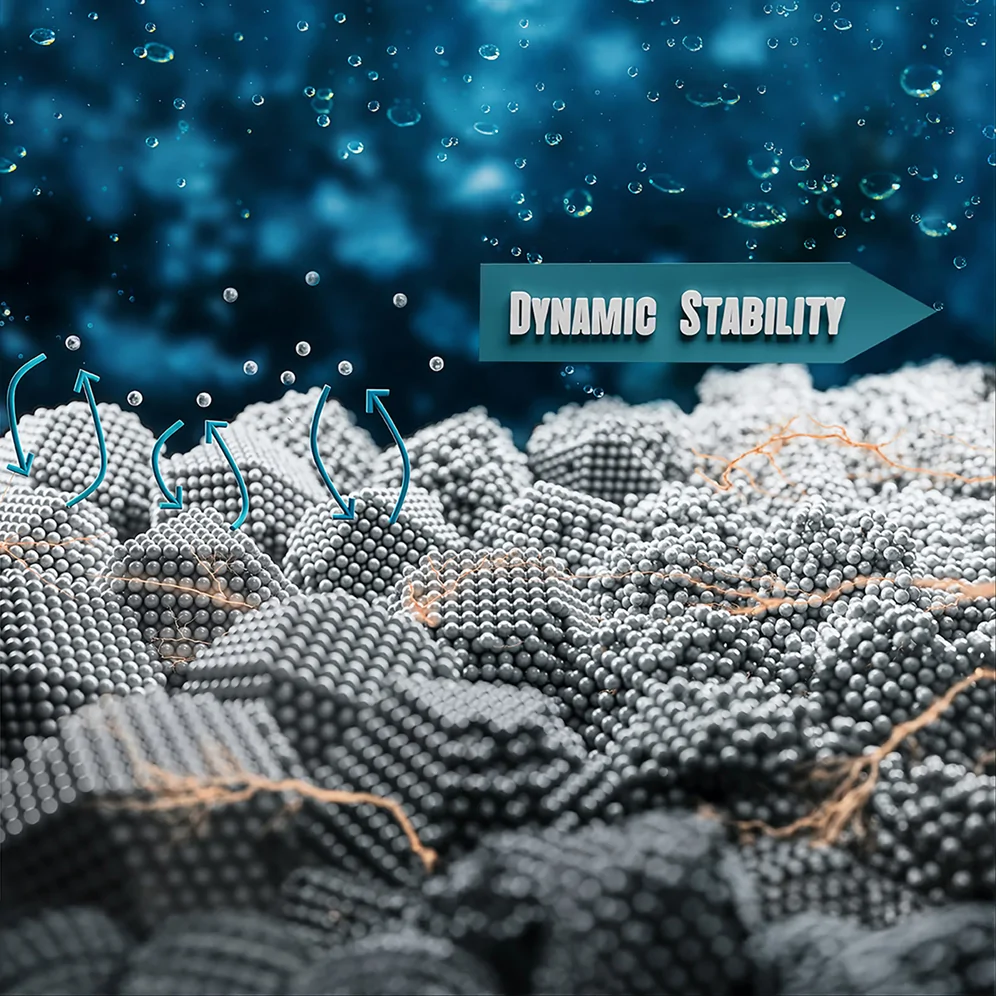

## Teaser

Metal electrocatalysts are often treated as static materials, yet they continuously exchange atoms with their surroundings during operation. This perspective argues that dissolution, migration, and redeposition form a universal restructuring loop that governs activity, degradation, and even recovery, reframing catalyst stability as control of atom flow rather than prevention of change.

## Introduction

**Figure 1:**
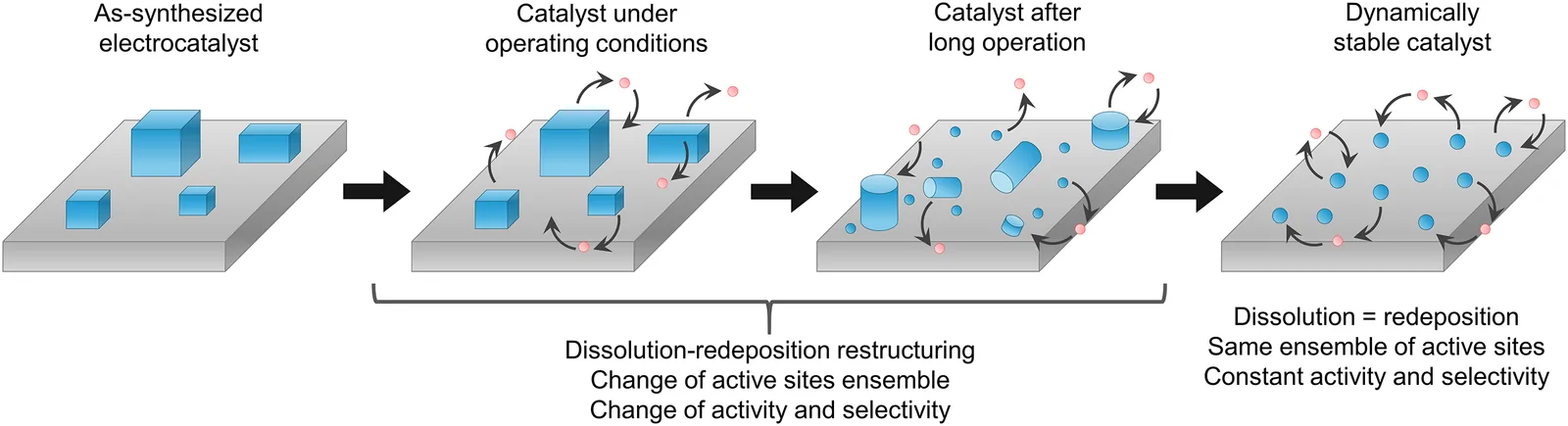
A schematic of catalyst reconstruction via dissolution redeposition under operating conditions

Electrocatalysis is central to the electrification of the chemicals and fuels industries, from water electrolysis and fuel cells to CO_2_ conversion and emerging electrosynthesis routes. In this landscape, “stability” is often treated as a material’s property: a catalyst is assumed to have a definable structure that persists under operation. Yet for most practical electrocatalysts, metals, and metal-derived materials, this static view is difficult to justify at the atomic level and especially on industrially relevant timescales.^1^^,^^2^^,^^3^^,^^4^ Under realistic electrochemical driving forces, atoms can leave the electrode, migrate through the electrolyte and porous catalyst layers, and reattach elsewhere.^5^^,^^6^ The result is not merely mass loss, it is surface evolution: active sites change, microstructures drift, and performance becomes history dependent rather than fixed (Figure 1).

A useful way to frame this behavior is to reconnect electrocatalysis with two older, well-established disciplines: corrosion science and electrodeposition. Corrosion is typically described as a coupled anodic-cathodic process, whereas electrocatalysis is often discussed through a single targeted half-reaction. Conceptually, however, both rely on the same elementary steps at the metal-electrolyte interface: oxidation/reduction of surface atoms, formation of soluble species, transport in the adjacent electrolyte, and (under sufficiently reducing conditions) redeposition. Electrodeposition and Ostwald ripening are governed by the same loop, even if the macroscopic outcomes differ (growth vs. degradation). In that sense, metallic cations and/or atoms “do not know” whether they are participating in corrosion, plating, ripening, or electrocatalyst evolution. The governing chemistry is the same, and what changes are the operating potentials, local environment, and boundary conditions imposed by the device and the application. These principles therefore extend well beyond electrocatalysis, underpinning processes across recycling, metallurgy, corrosion protection, sensing, energy conversion, energy storage, and related technologies.

This dissolution-transport-redeposition loop has two implications that are still unevenly appreciated across electrocatalysis. First, dissolution is frequently transient and coupled to surface redox transitions, potential cycling, and local chemistry. As a result, nominal “stability windows” inferred from equilibrium Pourbaix diagrams^7^ can be misleading for nanostructured catalysts under dynamic operation, where kinetic pathways, local pH gradients, adsorbates/intermediates, and evolving ion activities govern the interfacial speciation.^8^ A clear example is electrochemical CO_2_ reduction (CO_2_RR) on Cu at around −1.0 V vs. reversible hydrogen electrode (RHE): Cu would be classified as “stable” by Pourbaix-based intuition,^9^ yet numerous studies report pronounced restructuring^6^ and even detect measurable Cu dissolution at parts-per-billion levels during CO_2_RR.^10^ This apparent contradiction has been attributed to non-equilibrium interfacial conditions and stabilization/complexation of Cu species by CO_2_RR-derived ligands/intermediates,^11^ which shift soluble Cu speciation and lower the effective barrier for metal transport even under globally reducing potentials. Second, dissolution alone does not fully describe stability. Redeposition can mask net mass loss while still driving substantial restructuring, particle growth, porosity evolution, and metal redistribution within electrodes and membranes. Consequently, even small cumulative metal fluxes can reorganize electrochemically active surface area and site distributions, leading to time-dependent selectivity and efficiency.

The same processes that complicate catalyst stability are also directly linked to chemical circularity, for instance, through Pt dissolution and its recycling.^12^ Dissolved metals do not vanish; they enter an ionic inventory that can contaminate membranes, poison ionomers, nucleate new particles on supports, or deposit in unintended regions of an electrochemical device. Crucially, dissolution is often transient and coupled to specific electrochemical biasing, while its consequences accumulate over time as restructuring, ripening, detachment, redistribution within catalyst layers, or even staying in the membrane/electrolyte or the counter electrode of a device. Over long operating periods, even extremely low dissolution rates can accumulate and induce atomistic changes in catalyst structure and morphology, modify both the number and intrinsic activity of active sites,^13^ and ultimately result in measurable impacts on device performance (Figure 1). This matters not only for lifetime and reliability but also for end-of-life management: understanding where atoms go during operation is a prerequisite for designing lower-impact recovery routes and for closing material loops in electrified process technologies. Framed this way, electrocatalyst “stability” is not just a question of resisting change. It is a question of controlling pathways of atom flow to minimize harmful outcomes and, where possible, enable benign self-renewal.

A further complication is that restructuring is not always synonymous with failure, and not all restructuring observed under electrocatalytic conditions is caused by the catalytic reaction itself. In many systems, a substantial fraction of the initial structural evolution reflects precursor activation, including oxide reduction, hydration, or dissolution redeposition, during open-circuit exposure and the first applied bias.^14^^,^^15^ These transformations create the catalytically relevant surface, but they should be distinguished from reaction-conditioned restructuring, where the ongoing electrocatalytic reaction modifies dissolution and redeposition pathways through intermediates, adsorbates, and local interfacial chemistry. In systems such as Cu-based CO_2_RR, both contributions can occur sequentially and may even overlap, making this distinction essential for mechanistic interpretation.^10^ Dynamic evolution is therefore frequently intertwined with activation or “break-in,” where a precursor surface transforms into the catalytically relevant interface under operating potentials.^16^ In addition, some catalysts can evolve through more local solid-state pathways, such as oxide/(oxy)hydroxide growth, lattice oxygen redox, cation migration, or stress-induced amorphization, without dissolution redeposition necessarily being the dominant step.^17^

These insights motivate a reframing of electrocatalyst stability. Rather than asking whether a catalyst is structurally stable, it is often more productive to ask what the steady state of atom exchange is under a given operating protocol and whether that steady state preserves (or improves) performance. This perspective naturally leads to the concept of dynamic stability,^18^ where catalysts may remain functional not because atoms do not move but because dissolution and redeposition are balanced or steered in ways that maintain an effective population of active sites.^19^ In some cases, controlled transients (e.g., potential excursions and intermittent operation) may even be used intentionally to redirect restructuring into a more favorable morphology or composition, turning “instability” into a design variable rather than an uncontrolled failure mode.^19^^,^^20^^,^^21^

In this perspective, we do not simply revisit the now well-established view that electrocatalysts are dynamic under operating conditions, a point already emphasized in earlier perspectives on dynamic electrochemical interfaces, catalyst regeneration/self-healing, and broader function-stability relationships.^5^^,^^22^^,^^23^ Rather, we argue that dissolution-transport-redeposition offers a particularly powerful unifying framework for interpreting catalyst evolution across metals, reactions, and electrochemical devices. Our distinctive emphasis is therefore not only on restructuring itself but on atom flow: when and why atoms leave the catalyst, how they migrate through the local environment, where they redeposit, and how these cumulative processes reshape active sites over industrially relevant timescales. On this basis, we first outline the complementary experimental approaches needed to quantify these pathways across time- and length scales. We then examine case studies across different material classes and reactions, showing that corrosion, ripening, electrodeposition, activation, and electrocatalytic restructuring are deeply connected manifestations of the same interfacial chemistry. Ultimately, this perspective reframes catalyst stability not as the absence of change but as dynamic stability: the ability to maintain function while controlling the pathways of structural evolution.

## Experimental approaches

Capturing dissolution-transport-redeposition as a history-dependent phenomenon requires more than any single measurement: it demands experimental continuity and correlative datasets that link when atoms leave the catalyst, where they reappear (or vanish), and which chemical states and interfacial environments govern these pathways. In practice, restructuring depends not only on instantaneous potential, pH, and reactant identity but also on the full trajectory of synthesis, pre-treatment, device architecture, and electrochemical protocol, meaning that isolated snapshots can easily misclassify dynamic self-restructuring as “stability” or miss transient events that dominate long-term evolution. A data-driven framework therefore becomes an enabling methodology: by systematically preserving sample identity and experimental provenance and by integrating electrochemical response with time-resolved dissolution signals, identical-location (IL) microscopy, and *operando* spectroscopy, recurring patterns and mechanistic inconsistencies emerge directly from the combined evidence rather than from any one dataset alone.

Here, we highlight three complementary method families that—when deliberately linked—close the dissolution-redeposition loop across time and length scales. IL electron microscopy (IL-EM) provides spatially resolved, particle-by-particle evidence of restructuring and redistribution. Online flow-cell inductively coupled plasma mass spectrometry (ICP-MS) quantifies dissolution fluxes in real time and assigns them to specific electrochemical excursions. *Operando*/*in situ* spectroscopies constrain oxidation state, coordination, and adsorbates/intermediates that stabilize soluble species or gate redeposition, thereby connecting interfacial chemistry to atom transport. When these streams are stored in standardized, machine-readable formats with robust sample tracking (e.g., persistent identifiers/QR-linked records), they support iterative hypothesis refinement, traceability, and long-term reuse, turning restructuring from an interpretational ambiguity into a quantifiable, designable variable.

### IL-EM

**Figure 2:**
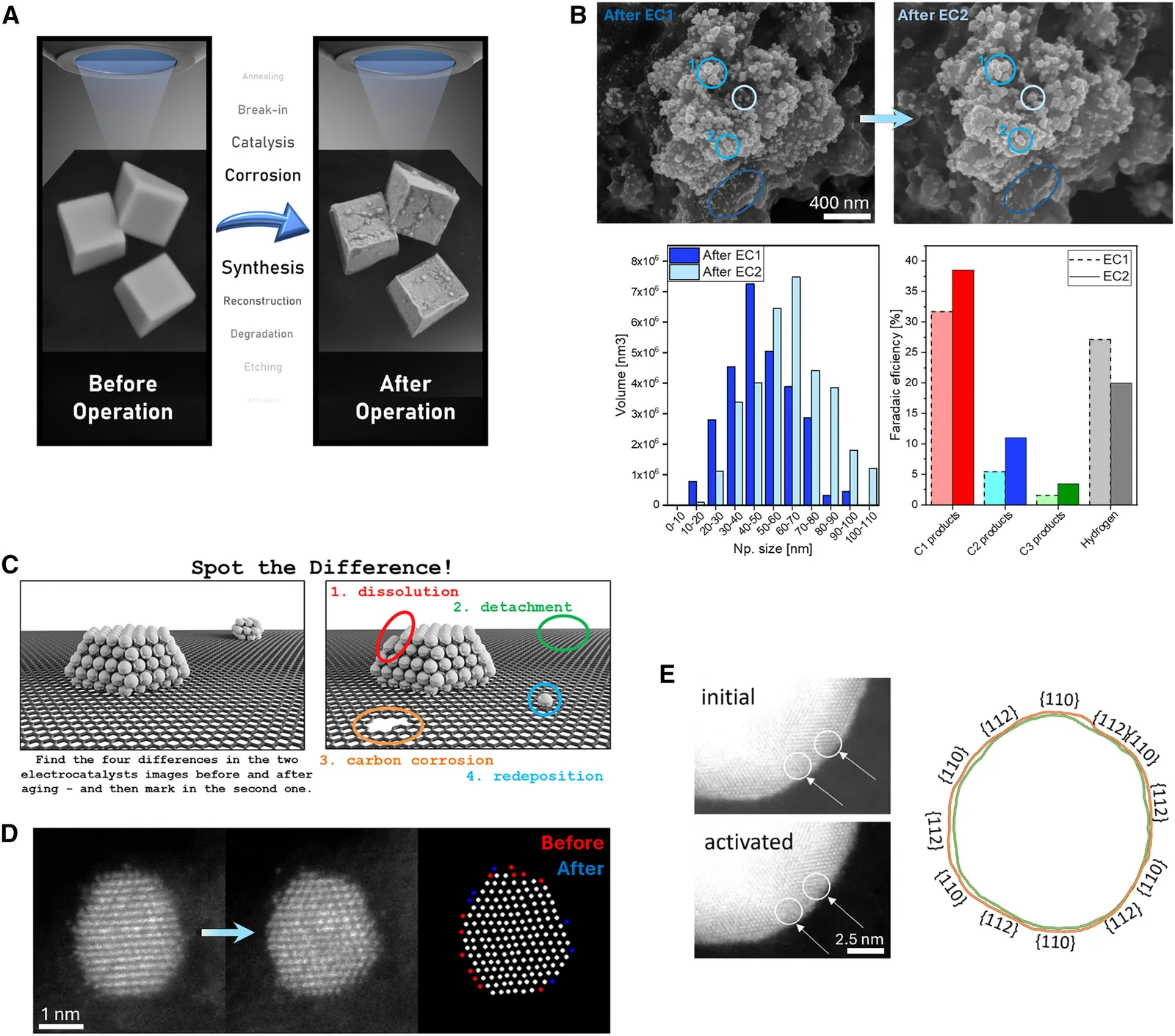
Identical-location electron microscopy reveals multiscale electrocatalyst evolution (A) Conceptual overview of the IL-EM workflow, in which the same catalyst regions are revisited before and after defined electrochemical histories to preserve spatial continuity from synthesis/break-in to catalysis and corrosion. Adapted from Tomc et al.,^24^ licensed under CC BY 4.0. (B) Representative IL-SEM images demonstrate how specific mesoscale features can be tracked across consecutive electrochemical steps, enabling quantitative analysis of structure-property relationships. Adapted from Tomc et al.,^24^ licensed under CC BY 4.0. (C) “Spot-the-difference” schematic summarizes key phenomena captured by IL-EM—dissolution, particle detachment, support/carbon corrosion, and redeposition—that together drive catalyst-layer restructuring. Adapted from Hodnik et al.^25^ (license number 6221310708148). (D and E) IL-STEM extends this approach to the nanoscale/atomic scale, resolving subtle transformations such as facet evolution, lattice/defect changes, and redeposition-induced growth within the same nanoparticle. Adapted from Hrnjic et al.^30^ and Kamšek et al.,^31^ licensed under CC BY 4.0.

To capture the intrinsically dynamic nature of metallic electrocatalysts under operating conditions, IL-EM can be used as a correlative strategy, enabling direct observation of structural and compositional evolution at precisely the same material regions before and after electrochemical exposure (Figure 2A).^24^^,^^25^^,^^26^^,^^27^^,^^28^^,^^29^ Unlike conventional postmortem or statistically averaged imaging approaches, IL-EM is specifically designed to follow individual particles, surface features, and local environments through electrochemical history, thereby resolving heterogeneous and often competing processes that would otherwise be obscured. This capability is particularly important for metallic electrocatalysts, which continuously undergo dissolution, migration, redeposition, and restructuring under reaction conditions, making any static description fundamentally incomplete. By establishing a one-to-one correspondence between electrochemical operation and localized material transformation, IL-EM provides a mechanistic framework to disentangle degradation pathways from adaptive or self-restructuring phenomena that directly affect catalytic performance.

Within the IL-EM framework, IL scanning EM (IL-SEM) offers a powerful means to investigate mesoscale and nanoscale morphological and compositional changes across extended electrode surfaces.^24^^,^^32^^,^^33^ By repeatedly imaging predefined regions of interest using stable markers and reproducible stage navigation, IL-SEM enables quantitative tracking of particle redistribution, surface roughening, void formation, and redeposition patterns with high statistical relevance (Figure 2B). The combination of secondary and backscattered-electron contrast, together with integrated energy-dispersive X-ray spectroscopy (EDS) and electron backscatter diffraction (EBSD), allows structural evolution to be directly correlated with compositional and crystallographic changes induced by electrochemical operation.^10^^,^^32^^,^^33^ Thus, IL-SEM is particularly well suited to visualize spatially resolved dissolution-redeposition dynamics and their coupling to electrode architecture, providing essential context for interpreting catalyst stability and activity trends (Figure 2B).

Importantly, integrating IL-SEM/EBSD/EDS with scanning electrochemical cell microscopy (SECCM) enables local electrochemical activity to be mapped directly onto precisely the same microstructural features, linking restructuring to spatially resolved catalytic response.^32^^,^^34^^,^^35^ Complementary time-of-flight secondary ion MS (ToF-SIMS) further extends this correlative approach by providing chemically specific information not only in two dimensions but also through depth-resolved, three-dimensional compositional mapping.^36^ In combination, these multimodal workflows bridge morphology, crystallography, surface chemistry, and local reactivity, enabling dissolution-redeposition processes to be resolved in both space and depth and directly connected to functional performance.

Complementary to IL-SEM, IL transmission EM (IL-TEM) extends the IL-EM concept to the nanoscale and atomic scale,^30^^,^^37^^,^^38^^,^^39^^,^^40^^,^^41^ enabling direct visualization of crystallographic, interfacial, and defect-level transformations within the same catalyst entities (Figures 2C–2E). By revisiting identical nanoparticles or thin regions before and after electrochemical treatment, IL-TEM allows subtle restructuring processes—such as lattice distortion, phase transformation, facet evolution, and redeposition-induced growth—to be resolved with high spatial resolution. This multiscale integration of IL-SEM and IL-TEM within a unified IL-EM framework provides a comprehensive picture of electrocatalyst dynamics spanning length scales relevant to both macroscopic performance and atomic-scale reactivity.

Images of catalyst sites taken at the same location provide a wealth of quantifiable information. This includes data on the sizes and shapes of catalytic particles and other distinct sites, their spatial distribution, the presence of defects, and various other features (Figure 2E). Careful analysis of these images, along with any accompanying spectral or other data, allows us to extract such information. Quantifying the features of interest in catalysts is well suited to automated data analysis, which has become an important and still evolving topic in the microscopy community.^31^^,^^42^^,^^43^ Images can be analyzed using conventional algorithms, machine learning approaches, or a combination of both.^44^ This variety of methods enables us to select and tailor an algorithm that is best suited for specific tasks, such as segmentation, connecting sites across datasets, or any other task aimed at enhancing our understanding of the sample being investigated.

In recent years, there have been multiple successful efforts to automate critical aspects of image and data analysis in IL-EM for electrocatalysts and beyond.^45^ Although machine learning techniques are increasingly becoming standard in this field, the requirement of providing sufficiently large amounts of labeled data to train supervised algorithms presents a significant barrier to entry. Nevertheless, with the emergence of freely available pre-trained models and other ready-to-use solutions, we can expect these approaches to be more widely adopted in electrocatalysis.^46^

A practical way to implement IL imaging as a life cycle experiment is the Nano Lab concept, which integrates catalyst preparation, electrochemical operation, and repeated IL characterization into a single transferable specimen.^47^ By preserving spatial and historical continuity, from synthesis to extended operation, the Nano Lab directly addresses a central theme of this perspective: connecting trace dissolution-redeposition fluxes to their consequences in space and time. In Nano Lab workflows, a TEM-grid-based working electrode can be operated in a modified floating electrode (MFE) configuration, where the grid is positioned at the electrolyte surface to enable electrochemical measurements at realistic current densities while minimizing artifacts from mass-transport limitations and gas bubble accumulation.^30^^,^^40^^,^^47^ Coupled with IL-TEM, this geometry allows restructuring pathways to be visualized rather than inferred; particle roughening and reshaping, agglomeration/Ostwald ripening, redeposition “hotspots,” and, in some systems, even single-atom generation and trapping on supports.

A similar visualization of changes induced by dissolution-redeposition dynamics can also be obtained by other microscopy techniques. In this sense, techniques such as atomic force microscopy (AFM)^48^^,^^49^ and scanning tunneling microscopy (STM)^50^^,^^51^ can be natural additions/replacements of the IL-EM workflows.

### Online ICP-MS

Online electrochemical flow-cell ICP-MS has become the workhorse method for quantifying electrocatalyst dissolution because it turns metal loss into a time-resolved signal that is synchronized with the potential/current history. In a typical setup, electrolyte continuously flows across the working electrode and carries dissolved metal species directly into the ICP-MS, enabling detection down to trace (sub-ppb) levels while running realistic electrochemical protocols.^52^^,^^53^^,^^54^^,^^55^^,^^56^^,^^57^^,^^58^ This is exactly the kind of “missing axis” needed for resolving the dissolution-transport-redeposition loop, i.e., when atoms leave the catalyst and at what rate, rather than inferring instability only from before/after morphology.

**Figure 3:**
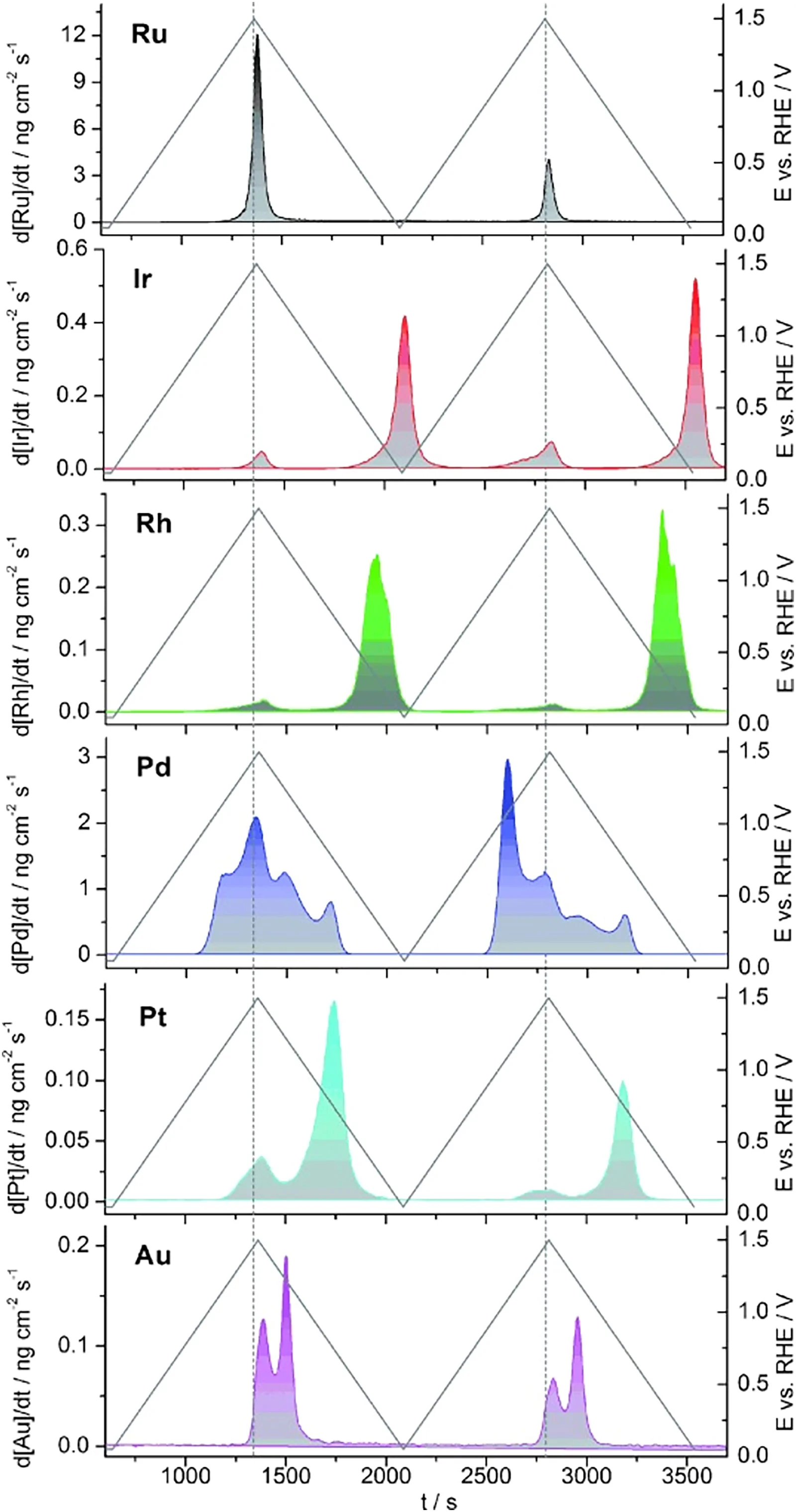
Potential-resolved, transient dissolution fingerprints from online ICP-MS Time-resolved dissolution rates (dM/dt) recorded during potential cycling show metal-specific dissolution bursts for Ru, Ir, Rh, Pd, Pt, and Au, highlighting that dissolution often occurs as sharp transient events associated with surface oxidation/reduction rather than as a steady background process. Adapted from Cherevko et al.^59^ (license number 6221310135625).

Crucially, online ICP-MS revealed that dissolution is often highly transient,^8^ with sharp peaks tied to specific electrochemical events, most prominently oxide formation and oxide reduction (Figure 3), even when classical thermodynamic intuition would suggest apparent stability.^59^ These potential-resolved dissolution fingerprints have been established across noble metals and conditions, and they explain why “average” mass loss can look small while restructuring is still significant: a sequence of short dissolution bursts can drive long-term redistribution, ripening, and changes in active-site populations. In addition to the dissolution mechanism, the impact of electrolyte and/or contaminants can also be studied using this technique. For example, the negative impact of Cl^−^ and methanol on the stability of metal electrocatalysts was revealed using an online ICP-MS setup.^60^^,^^61^^,^^62^^,^^63^

Because it is quantitative, the approach also enables comparative metrics (e.g., normalizing dissolution to charge passed or product formed^64^) and has begun to bridge model systems and device-relevant conditions.^65^ At the same time, ICP-MS on its own reports only what successfully leaves the electrode region as soluble species; it does not directly “see” redeposition or where the dissolved atoms end up. In this context, tracer-based approaches provide uniquely direct insight into dissolution-redeposition interplay. Isotope-labeling studies, particularly in Fe-containing oxygen evolution reaction (OER) catalysts, have demonstrated rapid and continuous exchange between dissolved and surface-bound species, confirming that dissolution and redeposition occur simultaneously and establish a dynamic steady state at the interface.^18^ These findings highlight that, in some cases, electrocatalyst stability should be understood not as the absence of dissolution but as the balance between dissolution and redeposition fluxes under operating conditions. For many electrocatalytic systems, redeposition remains experimentally challenging to observe directly and is therefore typically inferred from complementary techniques such as time-resolved ICP-MS, microscopy, and *operando* spectroscopy.

### Spectroscopic techniques

While online ICP-MS quantifies when and how rapidly atoms leave the electrode region, it does not reveal the chemical form they adopt during transport or after redeposition. *Operando*/*in situ* spectroscopy closes this gap by tracking oxidation state, phase, coordination, and adsorbates/intermediates under bias—i.e., the chemistry that often triggers dissolution bursts and governs whether dissolved species redeposit as metal or oxide/(oxy)hydroxide or become stabilized as soluble complexes.^66^^,^^67^^,^^68^ This is essential because “loss of metal” and “loss of function” are not synonymous: even modest dissolution fluxes can drive large structural drift if redeposition proceeds through a different chemical state.

**Figure 4:**
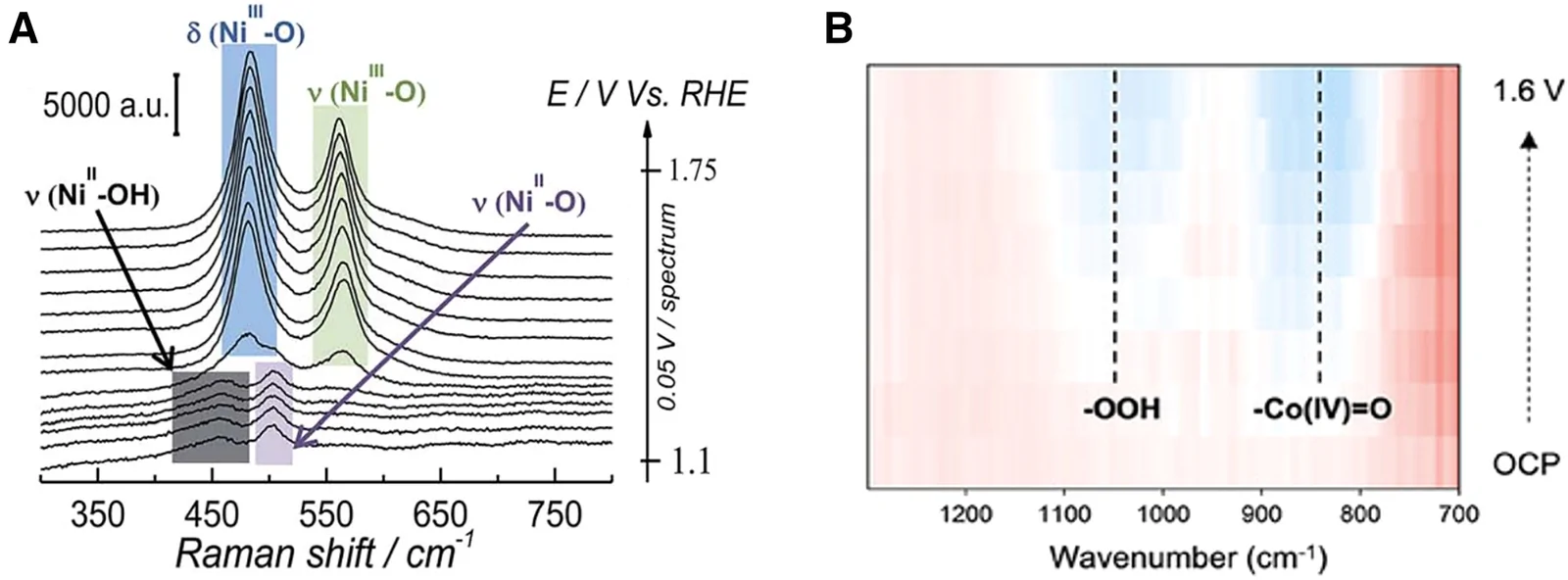
*Operando* vibrational spectroscopy reveals potential-driven surface oxidation during OER (A) *In situ* Raman spectra of Ni(OH)_2_/NiOOH during anodic polarization show the Ni^2+^/Ni^3+^ transition and the evolution of Ni–O vibrational modes associated with NiOOH formation and surface deprotonation under OER-relevant potentials. Adapted from Diaz-Morales et al.,^68^ licensed under CC BY 3.0. (B) *Operando* Fourier transform infrared (FTIR) of Co-based OER catalysts highlights the emergence of –OOH and Co(IV)=O species. Adapted from Li et al.,^73^ licensed under CC BY 4.0.

*Operando* Raman spectroscopy is a particularly accessible entry point because it sensitively detects surface (hydro)oxides, oxyhydroxides, carbonates/hydroxides, and reaction intermediates that accompany (and often control) dissolution-redeposition dynamics (Figure 4A).^69^^,^^70^^,^^71^ Since metals are intrinsically Raman inactive, Raman spectroscopy becomes especially informative when catalysts form Raman-active surface phases during operation or when signal enhancement is used. To overcome weak scattering and low coverage, the Raman signal is often enhanced using surface-enhanced Raman spectroscopy (SERS) or shell-isolated nanoparticle-enhanced Raman spectroscopy (SHINERS),^69^^,^^70^^,^^72^ which can enable detection of thin surface layers and transient intermediates under realistic electrochemical conditions. For example, Raman spectroscopy can follow the potential-driven formation of NiOOH or CoOOH during OER (Figure 4A), thereby linking phase evolution to activity and stability trends.^72^^,^^73^^,^^74^ For noble metals, *operando* Raman/SHINERS has been used to identify dynamic oxide/(hydro)oxide-like surface states on Ir and Pt^70^^,^^71^^,^^75^ and to capture transient high-valent features on RuO_2_ that can disappear as soluble species form.^76^^,^^77^ Cu is a special case in which the surface can be inherently SERS active when nanostructured: during CO_2_RR, oxidation dissolution followed by cathodic redeposition roughens Cu and creates hotspots, enabling *operando* tracking of surface oxides, interfacial carbonate/hydroxide, and adsorbed intermediates as restructuring proceeds.^50^^,^^69^^,^^78^

Beyond Raman spectroscopy, several complementary *operando* spectroscopies strengthen dissolution-redeposition interpretation by constraining chemical state and speciation under bias. X-ray absorption spectroscopy (XAS) can quantify potential-dependent changes in oxidation state and local coordination environment, helping distinguish true phase transformations from transient redox states that precede dissolution.^79^ X-ray photoelectron spectroscopy (XPS) provides surface-sensitive composition and oxidation-state information, making it valuable for tracking the emergence or removal of surface oxides and adsorbate-induced chemistry that can gate dissolution and redeposition.^80^ *Operando* infrared (IR) spectroscopy directly probes adsorbates and reaction intermediates that can stabilize dissolved metal ions or modify interfacial fields, thereby linking interfacial chemistry to metal transport (Figure 4B).^73^^,^^81^ *Operando* ultraviolet-visible (UV-vis) spectroscopy can access the electrolyte phase by monitoring soluble complexes and ionic species in real time, providing a direct handle on the “transport” part of the loop that is invisible to purely surface-based probes.^82^

Although electrochemical impedance spectroscopy (EIS) is not a spectroscopic technique in the classical sense of probing molecular vibrations or electronic transitions, it can be viewed as a frequency-domain spectroscopic method that resolves electrochemical processes occurring at different characteristic timescales. *Operando* EIS provides a kinetic and interfacial complement to these structural and spectroscopic tools by resolving charge-transfer resistance, double-layer capacitance, and mass-transport kinetics.^83^ Because dissolution redeposition alters active surface area, interfacial fields, and local transport pathways, it leaves distinct signatures in impedance spectra, enabling dynamic restructuring to be tracked through changes in reaction kinetics and interfacial capacitance. When interpreted alongside complementary techniques, *operando* EIS can link chemical state and restructuring to functional consequences, completing the feedback loop between structure, chemistry, transport, and catalytic performance.^19^^,^^84^

## Metals and mechanisms

### Pt dissolution and redeposition

Pt, a scarce and expensive noble metal, is most likely the most intensively studied electrocatalyst, particularly for the hydrogen evolution reaction (HER) and the oxygen reduction reaction (ORR) in acidic media, where it serves as the benchmark for activity and stability and plays a pivotal role in efficient green hydrogen production and fuel cell technologies. Although Pt is generally considered stable, it has been shown to undergo measurable dissolution even at the operating potentials of proton-exchange membrane (PEM) fuel cells, where, according to the Pourbaix diagram, it should remain thermodynamically stable.^85^ Moreover, pronounced Pt dissolution occurs under repetitive oxidation-reduction cycling in both acidic and alkaline environments, a phenomenon commonly referred to as transient dissolution.^86^^,^^87^

Pt dissolution into the electrolyte can be measured directly using online ICP-MS. However, this technique does not reveal how much of the dissolved Pt is subsequently redeposited. Such information can, to some extent, be obtained using IL-TEM or versions of this method. By enabling direct, particle-by-particle comparison before and after potential cycling, IL-TEM provides clear experimental evidence of Pt loss, migration, and growth that cannot be obtained from ensemble-averaged techniques such as statistical analysis of random TEM images. This method has been particularly valuable for linking local structural changes to electrochemical history, thereby offering direct insight into Ostwald ripening as a key dissolution-redeposition degradation mechanism in Pt-based electrocatalysts.

**Figure 5:**
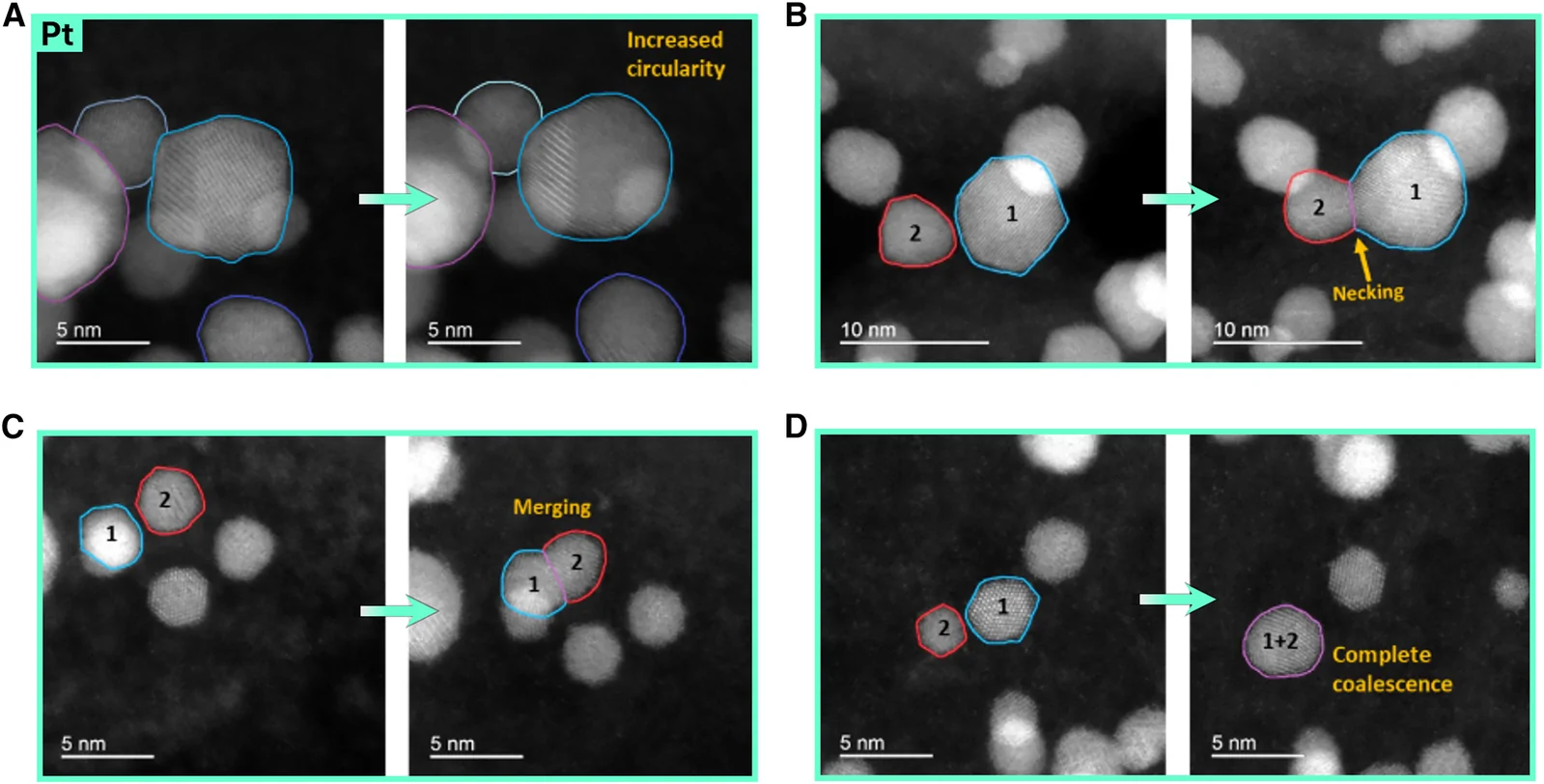
IL-TEM images of Pt nanoparticle restructuring (A–D) IL-STEM of different restructuring mechanisms governing changes of the PtCo nanoparticles induced by ADT performed at 60°C. Adapted from Matošin et al.,^94^ licensed under CC BY 4.0.

For example, IL scanning transmission microscopy (IL-STEM) has become an established method for locally probing diverse degradation mechanisms at the atomic scale in Pt-based electrocatalysts.^88^^,^^89^^,^^90^ Dissolution and redeposition of individual atomic columns were studied before and after potential cycling for carbon-supported Pt-Co,^30^ Pt-Ni,^91^ and Rh-Pt nanoparticles.^92^ More recently, the method was extended to four-dimensional STEM (4D-STEM), and the developed IL 4D-STEM (IL-4D-STEM) approach was used to investigate the relationship between the local dissolution of surface atoms and the underlying crystal structure in carbon-supported Pt-Cu nanoparticles.^31^ Combined with an elevated-temperature liquid half-cell accelerated degradation test (ADT) setup, IL-STEM enables direct visualization of the mechanisms by which Pt dissolution and redeposition reduce electrocatalyst active surface area and intrinsic activity.^93^ Tracking morphological changes in Pt nanoparticles at ILs showed that elevated-temperature ADT drives the system toward lower net surface energy, as reflected by the increased circularity of nanoparticle outlines (Figure 5A).^94^ Both dissolution and redeposition contribute toward this phenomenon, as protruding sharp edges of the Pt nanoparticles become more rounded through dissolution, while the concave surfaces are filled in through redeposition.

Another process in which dissolution and redeposition potentially play a crucial role is coalescence. If neighboring nanoparticles are close enough and their facets are aligned, they can become connected with a narrow bridge (Figure 5B). This process, commonly referred to as necking, represents an intermediate stage in the merging of two particles. Mass transport to the contact point between two nanoparticles is induced by the high surface energy resulting from negative surface curvature.^95^ The region is thus filled through the redeposition and/or atomic diffusion of Pt in order to minimize the net surface area, culminating in the formation of a neck.^96^ Further stages of coalescence result in the gradual merging and reshaping of the nanoparticles until they have completely coalesced into one larger, more spherical particle (Figures 5C and 5D).

Despite its high spatial resolution and chemical sensitivity, IL-TEM inherently probes a two-dimensional projection of a three-dimensional catalyst structure. As a result, the observed ripening behavior is restricted to surface-projected particle populations, and the apparent mass transport, coalescence probability, and growth kinetics correspond to a two-dimensional manifestation of Ostwald ripening. While this perspective is highly informative for understanding local dissolution-redeposition processes, it does not fully capture the redistribution of Pt within the volume of porous catalyst layers under realistic operating conditions.^85^^,^^97^

Three-dimensional Ostwald ripening involves Pt dissolution into the electrolyte, transport through the catalyst layer and ionomer network, and redeposition at spatially separated sites across the catalyst depth. Investigating this process requires access to statistically relevant particle ensembles and sensitivity to particles that are not directly captured in TEM projections. Although IL approaches have been extended to the device level,^37^ they still lack three-dimensional insight into degradation throughout the catalyst layer. These requirements exceed the practical limits of IL-TEM and necessitate complementary experimental approaches. In this context, IL-SEM is a natural extension of the IL methodology. IL-SEM enables repeated imaging of the same regions over large areas and at higher catalyst loadings while providing access to catalyst-layer-relevant length scales. This allows three-dimensional ripening effects to be probed more directly than is possible with TEM alone. Used together, IL-TEM and IL-SEM provide a consistent experimental framework for distinguishing between two-dimensional and three-dimensional ripening behavior and for developing a more complete mechanistic understanding of Pt dissolution and redeposition.^25^^,^^98^

### Ir behavior under OER

Ir, one of the scarcest noble metals, is most intensively investigated as a catalyst for the OER in acidic media, which constitutes the main energetic bottleneck in the production of green hydrogen. Despite its rapidly increasing price, Ir remains the benchmark OER electrocatalyst, as it offers the most favorable compromise between activity and stability under relevant reaction conditions. In our recent minireview, we discussed Ir dissolution, demonstrating that it is mechanistically linked to the OER via shared reaction intermediates and is strongly dependent on the structure of the Ir-based catalyst.^99^ When dissolution behavior derived from aqueous model systems was compared to degradation observed in real devices, significant discrepancies emerged, which we encouraged the community to address in future studies. At the device level, Ir degradation in PEM water electrolyzers can be quantified indirectly by tracking Ir redistribution within the membrane electrode assembly (MEA), particularly via accumulation in the membrane, catalyst layers, and water lines. This has been achieved either by ICP-MS analysis of digested anode and cathode layers and water loops or by postmortem SEM/STEM-EDS analysis of membrane cross-sections.^100^^,^^101^ These studies show that device-level stability is largely governed by the operating potential. Consequently, durability improvements at the device level should primarily be addressed through architectural, materials, and cell-level engineering strategies aimed at optimizing interfacial area, mass transport, and ohmic efficiency.

Nevertheless, identifying the fundamental drivers of Ir dissolution remains essential, as these ultimately define the limits of performance optimization. A key factor controlling Ir dissolution is the catalyst structure, which dictates both the accessible potential window and the dominant dissolution pathways. Importantly, the surface responsible for both high OER activity and enhanced dissolution is not an ordered crystalline phase but rather an amorphous or hydrous Ir oxide layer that forms dynamically under electrochemical polarization. This behavior was first identified on metallic Ir surfaces and later extended to crystalline IrO_2_,^102^^,^^103^^,^^104^ demonstrating that amorphization is a general and unavoidable feature of anodically polarized Ir. The formation of this amorphous layer is promoted under non-steady-state conditions, rendering it particularly relevant for intermittent electrolyzer operation. Detailed insight into the growth-dissolution interplay of hydrous Ir oxide has recently been provided by Zlatar et al.,^105^ who showed that hydrous oxide formation inevitably induces Ir dissolution. Based on the model proposed by Pickup and Birss,^106^ hydrous oxide growth proceeds via the formation of a compact inner IrO_2_ layer through a place-exchange mechanism, followed by oxidation and hydration of an outer layer that remains weakly bound during cathodic excursions. By studying thin films of varying thickness, they demonstrated that oxide growth, dissolution, and loss of metallic character originate from the same underlying mechanism, namely the permeability and reducibility of the compact inner oxide layer. This led to the concept of a size-dependent tri-layer interface, in which the reducibility of the compact oxide governs both Ir dissolution rates and catalytic durability.

Finally, while Ir dissolution has been predominantly studied in acidic aqueous electrolytes due to its direct relevance for PEM water electrolysis, Ir plays a central role across a wide range of electrochemical technologies.^107^ Consequently, systematic investigations spanning acidic, neutral, and alkaline pH regimes, as well as studies in the presence of organic compounds, are emerging and are essential for developing a unified, application-relevant understanding of Ir stability.^108^^,^^109^^,^^110^ For water-based systems, online ICP-MS measurements showed that Ir dissolution is mostly stable in acidic and neutral media but increases in alkaline environments. The study found that in the potentials before the onset of OER, the dissolution of Ir is mostly hindered by kinetics, not thermodynamics, and that the presence of anions also influences the dissolution rate. More interestingly, they showed that during OER, the surface is mostly stable in acidic and neutral pH, while dissolution in alkaline conditions increases, as IrO_4_^2−^, which dissolves more easily, is formed.^108^

### Dissolution redeposition of other precious metals

Other precious metals, such as Au, Pd, Ru, Rh, and Ag, also find versatile applications in electrocatalysis. Like Pt and Ir, those metals are also susceptible to different degradation routes and undergo restructuring under electrocatalytic conditions.

**Figure 6:**
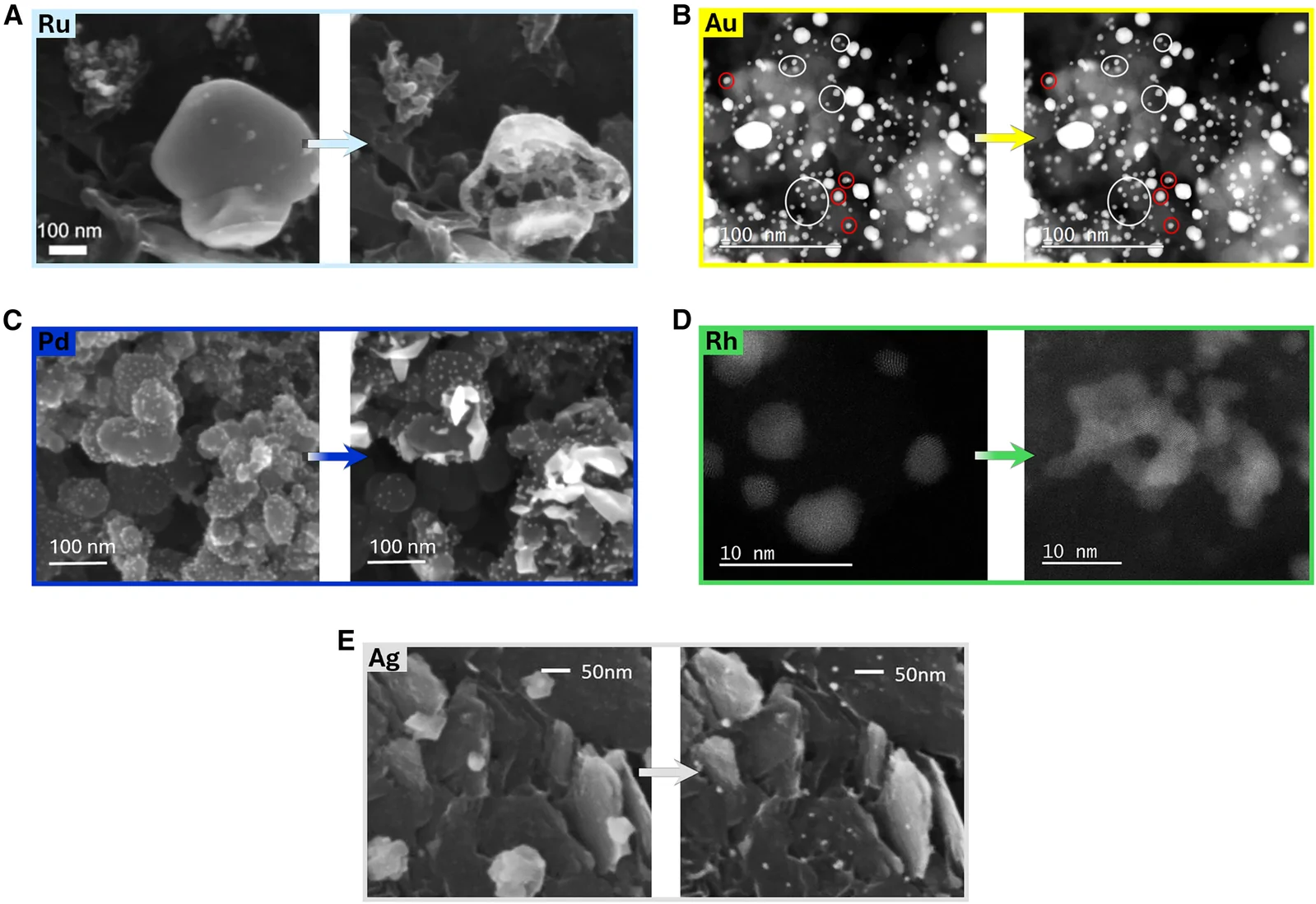
Morphological changes of noble metals (A) IL-SEM of metallic Ru particles before (left) and after (right) two cyclic voltammograms (CVs) between 0.05 and 1.6 V vs. RHE with a 1 V/s scan rate. Adapted from Hodnik et al.^112^ (license number 6221311110650). (B) IL-TEM imaging of a commercial Au/C sample before (left) and after (right) accelerated degradation test consisting of 10,000 CVs between 0.58 and 1.41 V vs. RHE at a 1 V/s scan rate. Adapted from Smiljanić et al.,^118^ licensed under CC BY 4.0. (C) IL-SEM imaging of a commercial Pd/C sample before (left) and after (right) accelerated stress test consisting of 3,000 CVs between 0.05 and 1.4 V vs. RHE at a 1 V/s scan rate. Adapted from Smiljanić et al.,^119^ licensed under CC BY 4.0. (D) IL-TEM imaging of a commercial Rh/C catalyst before (left) and after (right) accelerated degradation test consisting of 5,000 CVs between 0.05 and 1.4 V vs. RHE at a 1 V/s scan rate. Adapted from Smiljanić et al.,^120^ licensed under CC BY 4.0. (E) IL-SEM imaging of Ag nanoparticles before (left) and after (right) controlled cathodic corrosion at −10 V. Adapted from Vanrenterghem et al.^121^ (license number 6221311255983).

Ru-based electrocatalysts exhibit exceptional intrinsic activity for both the HER and OER, while they can be used to promote other reactions involved in fuel cells and electrolyzers (hydrogen oxidation, CO oxidation, methanol oxidation, etc.).^111^ The most attractive application of Ru-based materials would be to replace Ir as an anode catalyst in PEM water electrolysis, as RuO_2_ is intrinsically more active than IrO_x_ while being more abundant and less expensive. Under OER conditions, Ru readily undergoes electrochemical oxidation to form unstable high-valence species (e.g., RuO_4_ or soluble Ru(VIII)), leading to severe dissolution and loss of active material. Our group was among the first to study the dissolution behavior of nanoparticulated metallic Ru and Ru-oxide.^112^ Using an electrochemical flow cell coupled with ICP-MS (EFC-ICP-MS), it was revealed that Ru degradation proceeds through multiple dissolution pathways, including both thermodynamic anodic dissolution and extensive transient dissolution associated with changes in Ru oxidation state during potential cycling. In acidic media, dissolution occurs well below the nominal OER onset, with measurable Ru loss observed already at ∼1.23 V vs. RHE, which is approximately 0.17 V before the onset of significant oxygen evolution. At higher potentials (>1.4 V vs. RHE), extensive corrosion occurs via formation of unstable high-valence species (e.g., RuO_4_ and soluble Ru(VIII) compounds), leading to rapid material loss and particle disintegration. IL-SEM confirmed fast and severe dissolution of metallic Ru, which can be visualized in Figure 6A, whereas RuO_2_ exhibited more stable features. The degradation rate strongly depends on Ru phase and structure, as metallic Ru dissolves roughly 10× faster than electrochemically formed amorphous RuO_2_ and ∼100× faster than thermally prepared rutile RuO_2_, highlighting the decisive role of crystallinity, defect density, and oxidation-state stability. Nevertheless, at OER-relevant potentials (above 1.4 V vs. RHE), all Ru samples showed intensive dissolution, preventing their practical application. No redeposition of dissolved Ru was observed in IL-SEM imaging, which can be attributed to the lack of sufficiently reducing cathodic potentials (reaching in the HER region) and the high chemical stability of dissolved high-valent Ru oxo-species. Another systematic study on the OER activity and stability of metallic Ru and RuO_2_ was conducted by Cherevko et al.,^113^ confirming that metallic Ru dissolves readily under OER conditions, preventing any practical applications in water splitting technologies. Although more stable than the metallic counterpart, RuO_2_ still shows a dissolution rate one order of magnitude higher than that of IrO_x_ as the reference catalyst. These degradation mechanisms impose fundamental limitations on the operational lifetime and economic viability of Ru-based electrodes, making stabilization strategies such as structural ordering,^114^ electronic tuning,^115^ and support engineering^116^^,^^117^ indispensable for scalable electrolyzer and energy-conversion technologies.

Au is often regarded as one of the most stable noble metals; however, the introduction of modern *operando* studies showed that Au-based electrocatalysts can degrade significantly under realistic electrochemical conditions. A particular breakthrough was the introduction of an EFC-ICP-MS, enabling insights into potential-resolved dissolution profiles. These studies showed that Au dissolution begins already at ∼1.2–1.3 V vs. RHE and follows two regimes. Namely, at lower potentials, dissolution is dominated by transient oxide formation-reduction cycles, whereas above ∼1.6 V vs. RHE, direct anodic dissolution becomes the primary degradation mechanism.^122^^,^^123^ Comparisons across pH reveal that Au dissolves far more extensively in alkaline media than in acidic media, whereas in acidic OER-relevant conditions, it dissolves even more readily than Pt, reflecting fundamental differences in oxide structure and reaction pathways.^61^ Translating these insights into realistic nanomaterials, the stability of carbon-supported Au nanoparticles was recently investigated by our group.^118^ Accelerated stress tests in acid media showed a pronounced loss of electrochemical surface area of Au/C despite only minor total Au mass loss, which sounds counterintuitive. Using a combination of electrochemistry, IL-SEM/TEM, and online ICP-MS, it was shown that degradation is driven by preferential dissolution of the smallest nanoparticles (<5 nm) within a largely inhomogeneous Au/C sample. The smallest particles dominate surface area but contribute little to total mass, accounting for the observed trends in the degradation test. The crucial role of IL-TEM in discerning such a degradation mechanism in a rather inhomogeneous commercial Au/C sample is exemplified in Figure 6B. Electrolyte composition critically governs Au stability, and even trace chloride levels dramatically accelerate dissolution by stabilizing Au-Cl complexes and suppressing oxide passivation.^60^^,^^118^ At higher chloride concentrations, dissolution becomes highly efficient and can be explored for controlled electrochemical leaching of Au. Moreover, chemically induced dissolution of Au has been demonstrated as ozone exposure drives the potential at the Au surface to ∼1.45–1.50 V vs. RHE under open-circuit conditions, triggering sustained dissolution even without applied external potential. In the presence of chlorides, continuous Au nanoparticle dissolution can be achieved using ozone-induced potential alterations.^124^ Together, these findings overturn the traditional view of Au as electrochemically inert. Instead, Au displays highly dynamic dissolution behavior, governed by nanoscale morphology, oxidation/reduction cycles, and electrolyte impurities. Although early works suggested a negligible impact of Au redeposition, it can be strongly dependent on applied potential windows, scan rates, and mass-transport conditions. This knowledge of electrochemical Au dissolution processes, especially the interplay between oxide chemistry and halide complexation, provides a strong foundation for predicting catalyst degradation and designing efficient, environmentally benign Au-recycling strategies.

Palladium is another precious metal with wide application in electrocatalysis, including fuel cells, water electrolyzers, and CO_2_RR. Pd is most well-known for its exceptional ability to electro-oxidize small organic molecules (formic acid, ethanol, and methanol) and its potential application in direct liquid fuel cells. Therefore, understanding the stability and degradation pathways of Pd and its nanoparticulated counterparts is very important for their practical applicability. Recent systematic studies reveal that Pd is far less stable than traditionally assumed, exhibiting pronounced dissolution and restructuring under realistic electrochemical conditions. Early work on Pd dissolution proposed that anodic dissolution was the dominant mechanism, which was then argued by many authors proposing that the transient dissolution effect cannot be neglected.^125^^,^^126^ To unambiguously address this, recent studies relied on coupling an EFC with ICP-MS and IL-EM. Pizzutilo et al.^127^ conducted a detailed EFC-ICP-MS study showing that anodic dissolution dominates at lower potentials; in contrast, excursion to higher potentials above 1.5 V vs. RHE reverses the trend, and cathodic transient dissolution becomes prominent. A recent study performed by our group reported systematic accelerated stress tests of a commercial Pd/C catalyst, showing extensive Pd dissolution under dynamic potential cycling, coupled with significant redeposition and particle growth.^119^ In the more narrow potential range between 0.4 and 1.4 V vs. RHE, rapid potential cycling induced severe loss of electrochemical surface area (down to ∼11% of the initial value after only 5,000 cycles) and substantial Pd dissolution detected by *in situ* EFC-ICP-MS. Corresponding IL-SEM analysis revealed that nanoparticle disappearance was coupled with the appearance of larger particles via three-dimensional Ostwald ripening. Extension of the lower potential limit (LPL) to 0.05 V vs. RHE produced very large Pd crystallites on the outer catalyst layer, as shown in Figure 6C, while the amount of dissolved Pd remained similar. This behavior demonstrates that Pd degradation is triggered by dissolution and then driven by the dynamics between dissolution and redeposition, which is highly sensitive to the applied potential window and mass-transport conditions. Consequently, Pd instability under realistic start-stop operating conditions, when sudden potential spikes occur, could present a critical bottleneck for its deployment in energy conversion devices.

Similarly, Rh has very versatile electrocatalytic properties coupled with chemical inertness. For instance, Rh can be used as a very efficient HER catalyst or co-catalyst,^128^^,^^129^ whereas there are some promising reports on the OER performance of Rh-based materials. Moreover, it has been reported that the addition of Rh to Pt-based catalysts can improve their performance in the electro-oxidation of small organic molecules and for oxygen reduction.^130^^,^^131^ Electrochemical dissolution of Rh was studied by the group of Mayrhofer, showing that Rh, considered an inert metal, dissolves already at potentials below 1 V vs. RHE.^132^ In the following publication, the same group used a scanning flow cell coupled with ICP-MS to study potential and time-resolved dissolution profiles of Rh. It was shown that cathodic transient dissolution triggers much greater material loss than anodic oxidation and that steady-state dissolution of an oxidized surface is much smaller than dissolution during potentiodynamic conditions.^133^ To provide more information on the dynamics between Rh dissolution and redeposition, the electrochemical degradation of a commercial Rh/C material was studied by combining the IL-SEM, TEM, and EFC-ICP-MS approaches by our group.^120^ Rapid potential cycling between 0.4 and 1.4 V vs. RHE did not result in notable material degradation, as only minor Rh dissolution was detected. This was explained by the fact that the LPL of 0.4 V vs. RHE does not effectively reduce previously formed Rh oxides. Extending the LPL to 0.05 V vs. RHE resulted in much greater decay of the electrochemical surface area of Rh coupled with higher Rh dissolution, which was still very low and thus insufficient to account for observed electrochemical features. Comprehensive correlation between electrochemical tests, EFC-ICP-MS, and microscopy analysis revealed that redeposition of Rh plays an important role in the overall degradation mechanism. This can be visualized in Figure 6D, which shows an *ex situ* TEM analysis of Rh particles before and after degradation, revealing the appearance of Rh nanoparticles with larger diameters due to the redeposition and interconnection of individual particles. Similarly to Pt, Rh is sensitive to the reduction of previously formed oxide, resulting in Rh dissolution and redeposition. Therefore, such behavior of Rh needs to be considered when designing Rh-containing materials for electrochemical energy conversion devices.

Ag exhibits versatile electrocatalytic properties combined with comparatively low cost and high electrical conductivity. For instance, Ag is widely recognized as an efficient catalyst for the electrochemical reduction of CO_2_, displaying high activity and selectivity toward CO production.^134^ Moreover, Ag demonstrates promising activity toward the ORR, particularly in alkaline media, where it represents a viable non-Pt alternative. In addition, alloying or coupling Ag with other metals (e.g., Pt, Cu, or Pd) has been shown to enhance activity and/or selectivity in reactions such as ORR,^135^^,^^136^ CO_2_RR,^137^ and the electro-oxidation of small organic molecules through electronic and bifunctional effects.^138^^,^^139^ Therefore, knowing the degradation dynamics of Ag is important for designing highly active and stable nanocatalysts.^140^ Recent studies have shown that electrochemical degradation of Ag nanocatalysts proceeds through a combination of potential-driven dissolution and morphology evolution, strongly coupled to surface oxidation and the electrochemical environment. In alkaline media, Ag oxidation leads to the formation of surface oxides (Ag_2_O/AgO) and soluble species such as AgO^−^ or Ag(OH)_2_^−^, with both anodic and cathodic transient dissolution detected by time-resolved ICP-MS measurements.^141^ Notably, dissolution is significantly enhanced during dynamic potential cycling, particularly upon reduction of previously formed surface oxides, indicating that oxide formation-reduction transitions govern material loss rather than steady-state polarization alone. Complementary IL-TEM investigations demonstrate that Ag degradation under electrochemical operation involves Ag redeposition and nanoparticle agglomeration/coalescence in parallel with electrochemical dissolution, highlighting the dynamic nature of the process. More recently, direct nanoscale imaging using combined electrochemical liquid-phase EM (EC-LP-EM)^142^ revealed that Ag nanostructures begin to dissolve once the system is switched to open circuit potential (OCP). The dissolution is driven by the coupling of Ag oxidation (Ag^0^ → Ag^+^) with the ORR and is further accelerated by galvanic interactions when Ag is in contact with more noble substrates such as Pt. Importantly, degradation initiates at electrically connected regions and proceeds rapidly during early equilibration, demonstrating that Ag can undergo significant material loss even under idle conditions. Our group has studied the durability of Ag nanoparticles during carbon–halogen bond activation using electrochemical tests, analytical tests based on an EFC coupled with atomic absorption spectrometry (AAS), and IL-SEM.^143^ Despite the good activity of Ag, metal dissolution was observed to coincide with the reduction peak potential for the reduction of carbon-halogen bonds. IL-SEM analysis revealed nanoparticle agglomeration, which was assigned to the particle migration. Overall degradation mechanisms involved dynamics between metal dissolution and subsequent coalescence, leading to a drop in electrochemical surface area. Our group has shown that controlled cathodic corrosion of Ag can be a way to prepare uniformly dispersed nanoparticles below 5 nm (Figure 6E). Application of sufficiently negative pulses (e.g., −8 to −12 V) breaks down electrodeposited or agglomerated Ag nanoparticles (tens to hundreds of nm) into uniformly dispersed nanoparticles below 5 nm and even single atoms. Importantly, ICP-MS analysis confirmed negligible metal loss at optimized potentials (≤−10 V), demonstrating that cathodic corrosion can be used to counteract agglomeration/coalescence and restore electrochemically active surface areas in supported Ag electrocatalysts, as well as for other metals, including Pt, Ni, and Pd.

### Ni, Co, and Fe dissolution redeposition

Fe, Co, and Ni are widely used as catalysts for the OER and HER in alkaline media; however, their stability is highly sensitive to electrolyte composition, temperature, and applied current density. Quantifying metal dissolution under realistic operating conditions remains challenging. ICP-MS is commonly employed, but its workflow typically requires the dilution of concentrated KOH electrolytes (e.g., to ≤0.1 M KOH). This dilution compromises fidelity to industrially relevant conditions by altering metal speciation, corrosion kinetics, and redeposition behavior, and it restricts online or *operando* dissolution measurements under high alkalinity and high current density.^144^ If dilution is applied to a sample of 6 M KOH 60 times, the dilution will compromise measurement accuracy. Mehdi et al.^145^ employed online ICP-MS at low alkalinity (0.05 M KOH) and reported minimal Fe dissolution. However, such conditions do not reproduce the behavior observed at the high KOH concentrations (typically ≥6 M) and current densities relevant to commercial alkaline electrolyzers.^146^ Consequently, dissolution data acquired under dilute conditions must be interpreted with caution when extrapolated to practical systems.^144^

Beyond bulk dissolution measurements, it should also be considered that dissolution and redeposition may already contribute to the earliest stages of Ni oxidation and restructuring. Although the initial oxidation of Ni is often described as a surface-limited process that forms monolayer or near-monolayer oxygen-containing species on well-defined Ni surfaces,^147^^,^^148^ alternative mechanistic descriptions have been proposed. In particular, several studies suggest that transient Ni dissolution followed by reprecipitation as Ni(OH)_2_ may accompany, or partially contribute to, hydroxide-layer formation.^149^^,^^150^^,^^151^ In this view, the oxidized surface does not evolve exclusively through direct in-place transformation of the metallic lattice but through dynamic exchange between the electrode and the adjacent electrolyte. The growth and restructuring of Ni (hydr)oxide layers may therefore reflect a balance among surface oxidation, metal dissolution, and local redeposition. This possibility is especially relevant under concentrated alkaline electrolytes and technologically relevant operating conditions, where interfacial transport, local supersaturation, and metal speciation effects become increasingly important.

Under such conditions, alkalinity, temperature, and current density jointly govern dissolution and redeposition kinetics. Increasing KOH concentration from 0.1 to 7 M and elevating the temperature to 50°C both negatively shift the corrosion potential of Ni(OH)_2_, indicating accelerated corrosion kinetics.^152^ In 6 M KOH, bulk Ni electrodes, approximately 40 ppb Ni can be detected after 6 h, whereas for mesoporous Ni, ∼200 ppb dissolved Ni was measured under identical conditions, suggesting that increased porosity compromises structural integrity and enhances dissolution.^153^ Under variable operation in a zero-gap alkaline electrolyzer using 7 M KOH, Ni dissolution during OER increased the dissolved Ni concentration from ∼106 to ∼503 ppb over 60 h.^152^ These results collectively demonstrate that both electrolyte severity and electrode morphology strongly amplify Ni dissolution under practical conditions.

**Figure 7:**
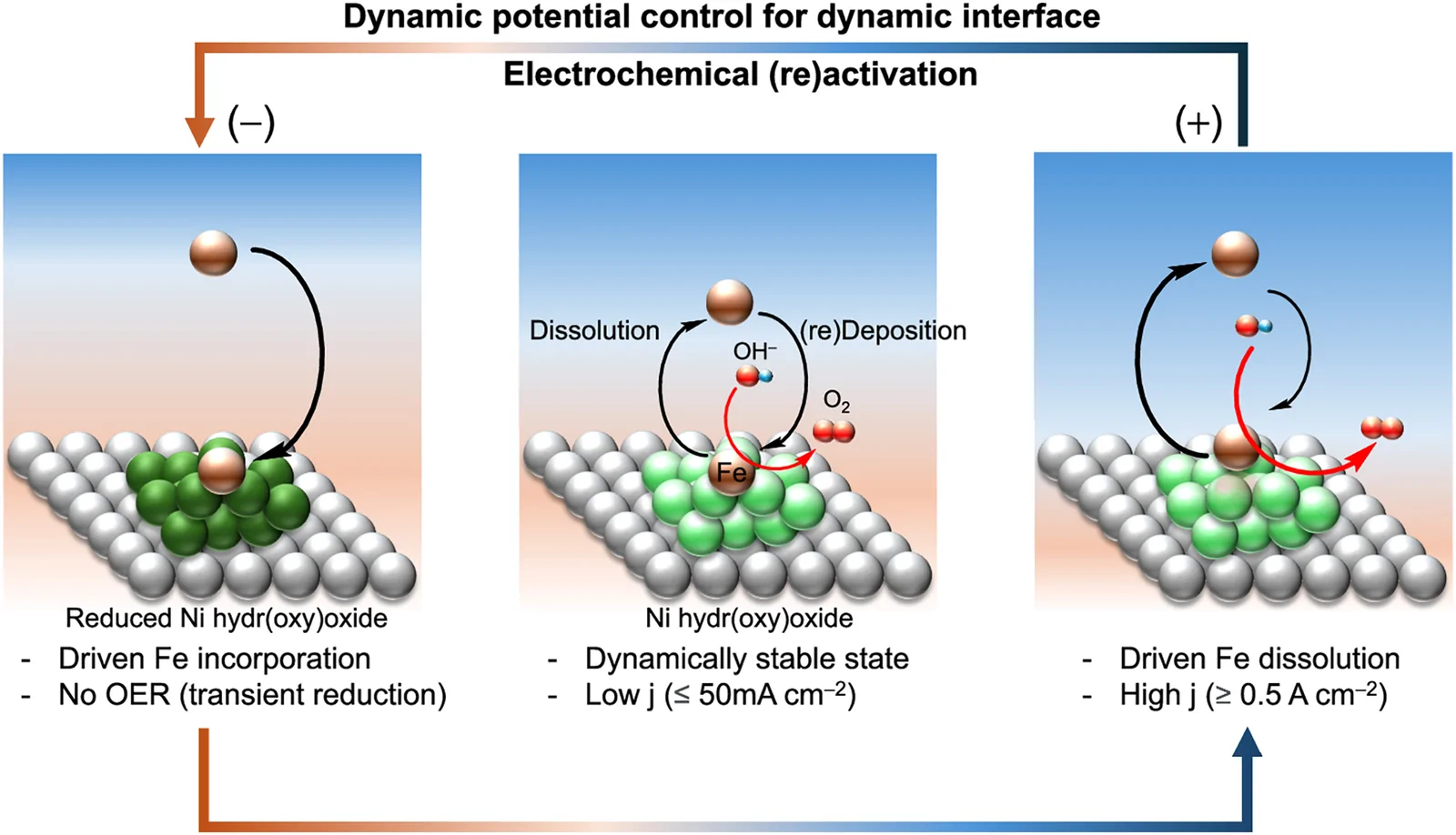
Dynamic stability of Fe under pulsed OER conditions Adapted from Han et al.,^157^ licensed under CC BY-NC-ND 4.0.

Fe dissolution exhibits a similarly strong dependence on electrolyte concentration. Speck et al. reported significantly higher Fe dissolution in 5.4 M KOH compared to 0.1 M KOH.^154^ The introduction of an anion-exchange membrane reduced Fe loss at the anode by suppressing Fe migration and redeposition at the cathode, indicating partial retention of Fe under steady-state operation. During potential cycling or reoxidation of surface oxide films, sharp Co dissolution spikes are observed, with smaller but analogous features reported for Ni. Notably, the presence of Fe ions in KOH suppresses Ni and Co dissolution,^155^ an effect consistent with lower Ni dissolution in NiFe(OH)_2_ compared to NiCo(OH)_2_ catalysts.^152^ Fe dissolution and incorporation dynamics are strongly conditioned by current density and Fe concentration in the electrolyte. At room temperature in 1 M KOH and under low current densities, Fe dissolution from the electrode can cause deactivation. However, if the electrolyte already contains dissolved Fe, a dynamic stability state is reached in which Fe circles from the electrode to the electrolyte and back without a net loss. Such a regime has been shown to sustain OER activity.^18^ In Fe-purified electrolytes, rapid deactivation of Fe-Ni, Fe-Co, and Fe catalysts is observed, underscoring the importance of sustained Fe availability. This was studied in detail by Chung et al.^18^ using an isotope-labeling approach. Specifically, Ni and Co hydr(oxy)oxide substrates were modified with ^56^Fe, while ^57^Fe was added to the electrolyte. Monitoring of the ^57^Fe/Fe ratio revealed that overall Fe content on the electrode remains stable during OER tests and that more than 50% of ^56^Fe was exchanged with ^57^Fe after only 1 min. This indicates that Fe dissolution and redeposition are very rapid processes that govern the reactivity of Fe-containing Ni- or Co-based anodes. Building on this approach, the following work, based on isotope tracing, demonstrated that Fe redeposition is not random but can be promoted by catalyst structure, where confinement and facet-dependent adsorption enable a localized dynamic equilibrium of Fe species at the interface.^156^ At higher current densities, Fe loss accelerates, necessitating active strategies such as dynamic polarization or deliberate maintenance of solution-phase Fe to retain catalyst composition (Figure 7).^157^ Conversely, periodic refreshing of the electrolyte with Fe-free solution results in continuous Fe depletion.^158^ The kinetics of Fe incorporation differ markedly between hosts: reported incorporation times to reach ∼8% Fe on CoOOH surfaces are ∼2 h, whereas NiOOH reaches ∼5% Fe within ∼10 min under identical conditions (η = 350 mV, 1 M KOH), as determined by XPS.^159^

While alkaline corrosion is often associated with anodic operation, substantial degradation can also occur under cathodic polarization. Standard Pourbaix diagrams have limited predictive power for electrode stability under realistic HER conditions because they neglect kinetic effects, cathodic corrosion phenomena, and the influence of temperature, pressure, and ionic activity.^160^ The cathodic corrosion of Ni is particularly significant. In 1 M KOH at room temperature, corrosion rates of ∼0.008 mm·year^−1^ have been reported, increasing to ∼0.096 mm·year^−1^ in 85% KOH at 160°C and 200 mA·cm^−2^.^161^ This corrosion is accompanied by surface restructuring, including loss of Ni(200) facets and growth of Ni(111), consistent with dissolution-redeposition mechanisms. Cathodic corrosion has also been observed in acidic media: in 0.5 M H_2_SO_4_ at 0.2 mA·cm^−2^, Ni corrosion rates were ∼5.2 × 10^−3^ mm·year^−1^, approximately 20 times lower than immersion-derived values, but increased markedly at higher current densities.^162^ Electrolyte impurities further exacerbate cathodic corrosion. Chloride ions enhance corrosion rates on steels,^163^ and for stainless steel SS304L, oxide reduction above pH ∼16.5 can dominate cathodic processes and drive corrosion.^164^ Stabilization strategies have therefore been explored: Jiang et al.^165^ demonstrated stabilization of α-Co(OH)_2_ during HER in 1 M KOH at −10 mA·cm^−2^ through the addition of MoO_4_^2−^, which was proposed to mitigate interfacial OH^−^ accumulation. During HER in 0.1 M NaOH, Branton and Coutanceau observed Co dissolution at −0.4 V vs. RHE, accompanied by solution discoloration, which they attributed to the formation of soluble Co(OH)_4_^2−^ species.^166^ Similar behavior was observed in Ni_x_Co_10-x_ alloys, although Co stability improved for x > 5. Importantly, dissolution rates scale nonlinearly with current density and hydroxide activity, meaning that extrapolation from laboratory conditions (≤100 mA cm^−2^, ≤1 M KOH) to industrial operation (>1 A cm^−2^, ≥6 M KOH) can underestimate degradation by orders of magnitude.

Microstructure is slowly revealing itself as a missing descriptor in electrocatalyst stability discussions: electrodes with identical bulk composition can evolve very differently simply because their grains, grain boundaries, defects, and secondary phases impose distinct local reaction environments and corrosion pathways. Recent grain-resolved work on Inconel 625 demonstrates this clearly by combining IL EBSD/SEM/EDS with localized electrochemistry, revealing that alkaline OER activation produces strongly heterogeneous, grain-dependent surface transformation and up to ∼30% variations in local OER activity across a single polycrystalline surface.^32^ In this study, the formation and roughness of the NiFe (oxy)hydroxide layer depended on grain size and microstructural features, with grain boundaries and fine-grained regions undergoing the most pronounced restructuring, consistent with microstructure, guided dissolution, and selective film growth.

More broadly, emerging evidence shows that surface reconstruction, dissolution redeposition, and active-phase formation are inherently spatially heterogeneous processes governed by local crystallography, defect density, strain, and coordination environment.^167^^,^^168^^,^^169^^,^^170^^,^^171^^,^^172^^,^^173^ In particular, grain boundaries (and related dopants) and microstructural defects introduce locally strained and undercoordinated environments that modify the electronic structure and provide distinct catalytic sites, directly linking microstructure to both activity and stability.^169^ Rather than forming uniformly, dynamically generated (oxy)hydroxide phases and defect-rich active states preferentially emerge and stabilize at specific microstructural sites, where local electronic structure and adsorbate interactions favor their persistence under operating conditions. In this view, stability and activation are not purely material-composition problems but microstructure-controlled phenomena in which atom fluxes, redistribution, and redeposition are intrinsically biased by the electrode’s grain architecture.

Such studies highlight an underexplored but practically critical point: “stability” and activation are not purely materials-composition problems but microstructure-controlled phenomena in which dissolution, redistribution, and redeposition are spatially biased by the electrode’s grain architecture.

### Cu dynamics under CO_2_RR and other electrosynthesis conditions

**Figure 8:**
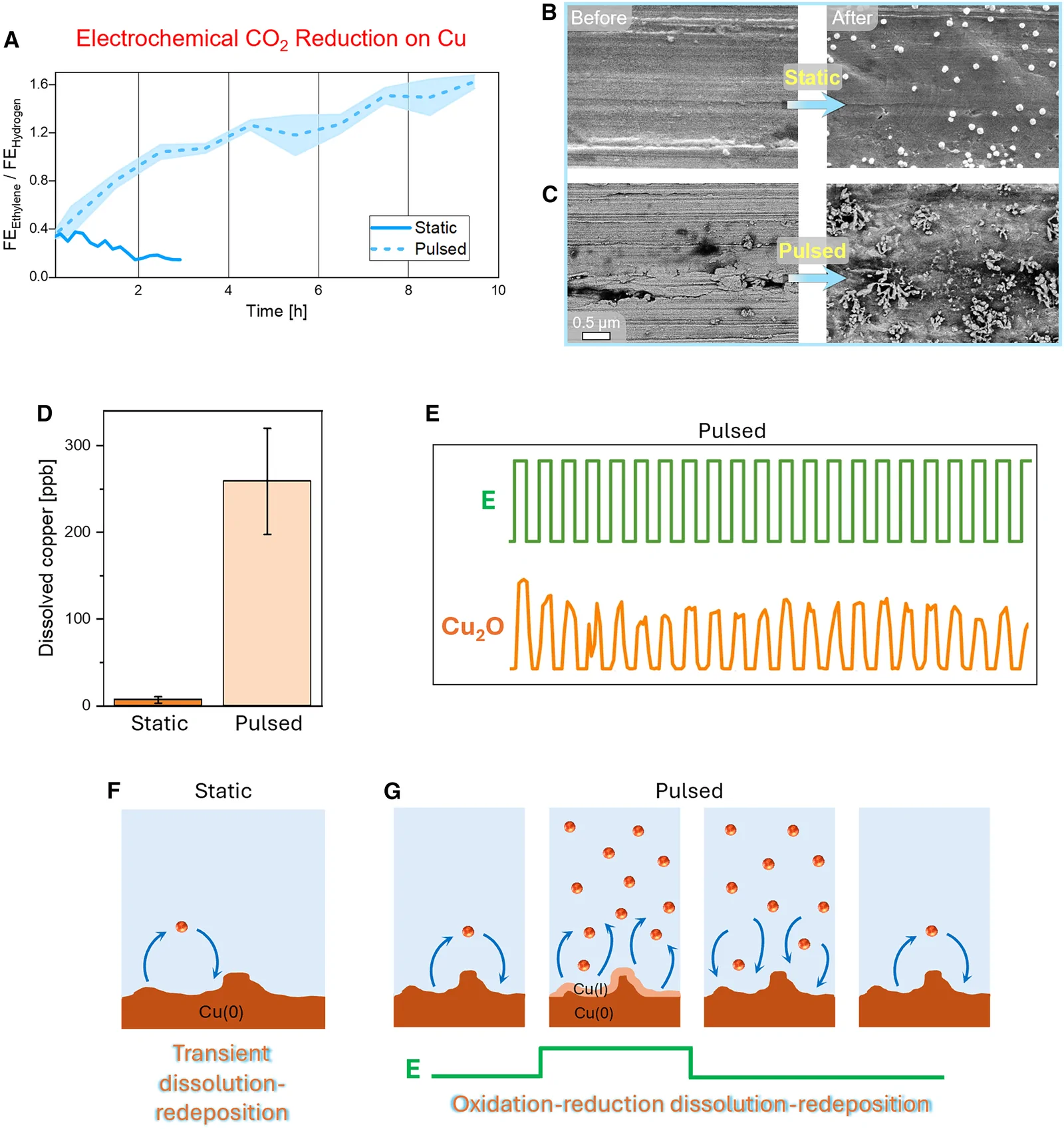
Cu dynamics under CO_2_RR and their control by electrochemical pulsing (A–C) The impact of static vs. pulsed electrolysis on (A) CO_2_RR performance evolution and (B and C) Cu catalyst morphology restructuring, highlighting fundamentally different restructuring pathways and selectivity outcomes. (D) Rate of Cu dissolution under the two different regimes. (E) *Operando* Raman spectroscopy under pulsed CO_2_RR uncovered the formation of Cu_2_O under the oxidative segment of the pulse. (F and G) Conceptual illustration of dissolution redeposition under (F) only a reductive CO_2_RR bias and (G) a pulsating electrolysis initiating Cu oxidation. (A)–(D) were adapted from Tomc et al.,^19^ licensed under CC BY 4.0. (E) was adapted from Herzog et al.,^179^ licensed under CC BY 4.0.

Cu is, to date, the only electrocatalyst enabling efficient CO_2_RR.^174^ While promising, it suffers from the gradual and severe morphological instability, which in turn hinders the selectivity of CO_2_RR (Figures 8A and 8B).^10^^,^^175^ The underlying process for this restructuring is a transient dissolution redeposition of Cu at ppb levels (Figures 8D and 8F).^10^^,^^176^ Since CO_2_RR occurs at −1 V vs. RHE, a potential deep in Pourbaix stability window of metallic Cu,^9^ the dissolution of Cu is counterintuitive. However, as Vavra et al.^11^ elegantly demonstrated, CO_2_RR intermediates must be considered to understand this process. In subsequent studies, it was shown that species such as CO can drive Cu leaching into the electrolyte and that their dynamic dissolution-redeposition processes drive morphological restructuring toward the formation of fewer CO_2_RR active sites.^177^ Importantly, such processes also occur in MEA cells.^178^

However, this is not the only dissolution-redeposition process of Cu reported in the literature. Notably, at the very start of the CO_2_RR reaction, Cu severely restructures.^14^ The mechanism was identified as Cu oxidative-reductive dissolution redeposition.^10^^,^^14^^,^^15^^,^^180^ Cu oxides present on the catalyst’s surface dissolve into the electrolyte under OCP conditions, following the Pourbaix diagram. When the potential is applied to initiate CO_2_RR, these dissolved Cu species redeposit onto the surface, forming distinct morphological motifs from their precursors.^14^ These active sites are differently active for CO_2_RR than the precursor, complicating the structure-property relationship for Cu-catalyzed CO_2_RR from the very start of the reaction.^14^ Crucially, the catalytic reaction already plays an active mechanistic role in this initial restructuring. In our recent work, Cu redeposition at the onset of cathodic polarization was compared under CO_2_-saturated and Ar-saturated conditions.^10^ Under Ar, where no CO_2_RR products were formed and only HER occurred, pre-existing Cu nanoparticles grew comparatively evenly, consistent with more classical curvature-driven growth/Ostwald-type behavior. In contrast, under CO_2_RR conditions, the redeposition became strongly anisotropic: reaction intermediates partially or fully blocked parts of the Cu surface, forcing dissolved Cu species to redeposit on less intermediate-covered regions instead. This shows that even initial Cu “activation” is partially driven by CO_2_RR. The resulting surface motifs then exhibit different CO_2_RR selectivity, linking reaction environment, restructuring pathway, and catalytic performance.

While on the one hand, oxidative-reductive dissolution redeposition complicates the understanding of CO_2_RR, on the other, this process can be used to *in situ* “override” the transient dissolution redeposition.^19^ This is achieved by electrochemical pulses, interrupting the applied reductive potential with an oxidative bias,^181^ facilitating *in situ* Cu oxidation-reduction dissolution redeposition (Figures 8D, 8E, and 8G). Importantly, recent studies have demonstrated that these potential excursions can stabilize CO_2_RR selectivity (Figure 8A).^182^^,^^183^ This is initiated by more than an order of magnitude higher Cu dissolution vs. a pure static regime (Figure 8D), completely suppressing the transient dissolution-redeposition behavior of Cu.^19^ By *in situ* controlling the dissolution redeposition with potential pulses, the morphological restructuring is different (Figure 8C), and this in turn favorably affects CO_2_RR selectivity (Figure 8A). Interestingly, such an environment could also be enabled by intentionally adding Cu to the electrolyte.^184^

The knowledge of Cu dynamics under CO_2_RR conditions is directly transferable to other Cu-catalyzed electrosynthesis processes, e.g., NH_3_ production from NO_3_,^20^ formaldehyde oxidation,^185^ etc.^186^

### Li dissolution redeposition under N_2_ reduction

**Figure 9:**
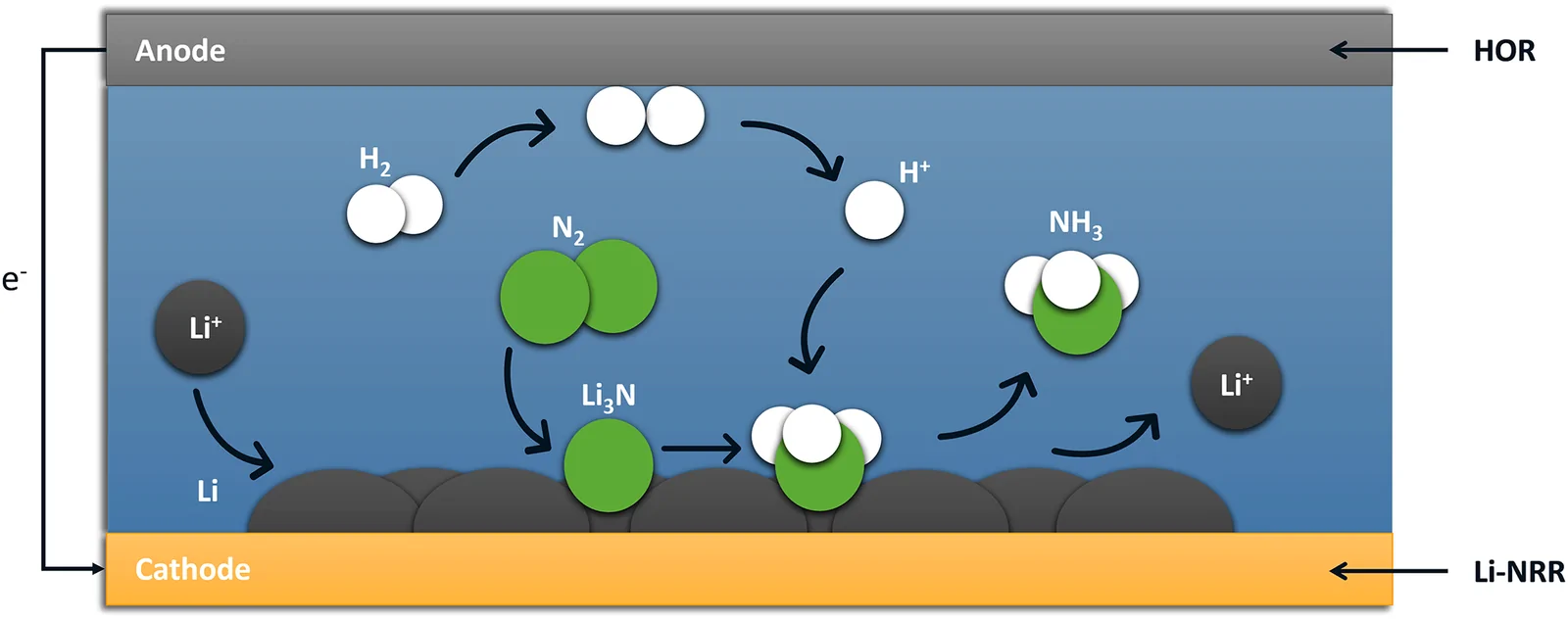
Schematic of the Li-NRR mechanism Side reactions and complex solid-electrolyte interphase (SEI) formation are neglected. Created by the authors based on the concept proposed by Chang et al.^197^

The only electrochemical method of NH_3_ synthesis reducing N_2_ unambiguously is the non-aqueous Li-mediated nitrogen reduction reaction (Li-NRR).^187^^,^^188^^,^^189^ The most prosperous Li-NRR electrochemical protocol switches between Li deposition potential and OCP periods, implementing the so-called pulsing protocol. Such a protocol results in significantly improved faradaic efficiency toward NH_3_ compared to the widely used constant current methods,^188^^,^^190^^,^^191^^,^^192^ partially due to the suppression of side reactions between the organic electrolyte and metallic Li.^191^^,^^193^^,^^194^^,^^195^^,^^196^ Instead, during the OCP periods, the metallic Li, deposited on an inert electrode (e.g., Cu or Mo) from dissolved Li salt, reacts with N_2_ gas to form Li_3_N, followed by a series of electron and proton transfers where NH_3_ forms in the presence of a suitable proton source (Figure 9). Consequently, the pulsing protocol was able to keep the Li-NRR electrode potential in a stable regime for days.^191^ The pulsing protocol (a transient regime) is crucial for electrochemical stability in Li-NRR operation. Although electrode stability under a transient regime is a major focus in aqueous electrochemistry owing to its relevance for fuel cells and electrolyzers, electrode dissolution in non-aqueous electrochemical systems has received little attention. This is likely to change as the Li-NRR field continues to develop, in which the cathode and anode potentials oscillate over time during Li-NRR operation.^188^^,^^190^^,^^191^^,^^192^ A stable cell voltage is obtained when the pulsing Li-NRR (cathodic reaction) method is combined with the hydrogen oxidation reaction (HOR) on a Pt-based catalyst, presenting the most realistic anodic reaction during NH_3_ production.^188^

According to recent studies monitoring Pt dissolution online, Pt dissolution under non-aqueous conditions fundamentally differs from that under aqueous conditions. Under potentiodynamic perturbation, only anodic dissolution of Pt takes place,^198^^,^^199^ contrary to the Pt dissolution mechanism observed in aqueous electrolytes.^200^ Therefore, operating under non-aqueous conditions mitigates the decrease in the active surface area and surface restructuring (Pt dissolution redeposition) that progressively lowers intrinsic catalytic activity observed in aqueous catalysis.^39^^,^^40^ However, Pt dissolution redeposition in non-aqueous electrolytes cannot be fully disregarded, as the surface Pt enrichment observed for the PtAu catalyst suggests that such a mechanism is present in the Li-NRR system.^188^ Furthermore, the HOR activity under a non-aqueous regime decreases rapidly with potential due to the adsorption of organic solvent and bulky electrolyte species blocking/poisoning the HOR.^198^^,^^199^ Therefore, any further mechanism leading to the loss of surface area (e.g., Ostwald ripening) would gradually increase the anode potential, triggering undesired oxidative degradation of the organic electrolyte.^192^ Moreover, the gradual build-up of dissolved Pt species in the electrolyte will drive their diffusion toward the cathode, ultimately leading to redeposition. Consequently, the selectivity for NH_3_ should certainly decline due to increased competitive HERs and potentially other parasitic reactions.

Li-NRR studies have predominantly focused on the solid-electrolyte interphase (SEI), as the diffusivities of Li^+^, N_2_, and proton donors through the SEI have been identified as the key factors governing the maximum attainable ammonia selectivity.^190^^,^^195^ In contrast, the nature of the electrode substrate has scarcely been investigated. However, a recent study on Cu(111) single-crystal substrate revealed restructuring toward polycrystallinity under Li deposition potentials, suggesting that restructuring is caused by Li plating.^201^ According to battery studies, Li can diffuse into Cu electrodes, where incorporated Li atoms serve as nucleation sites for subsequent Li deposition. This process may induce surface roughening and structural reorganization of the Cu substrate, facilitated by the high surface mobility of Cu atoms.^202^ Increased surface roughness is expected to hinder uniform Li deposition and favor dendritic growth, which can alter the morphology of the SEI, hence likely influencing the efficiency of Li-NRR. Overall, the restructuring of the Cu substrate suggests interactions with Li salt under Li-NRR conditions. However, their direct impact on Li-NRR performance remains unresolved. Further restructuring of the Li-NRR electrode substrate (e.g., Cu) can reasonably be anticipated due to the so-called cathodic corrosion at extremely negative potentials, i.e., an electrochemical etching process that forms a range of metallic surface morphologies.^203^^,^^204^ In terms of Li-NRR, the extent of the restructuring is likely to be even larger due to the pulsing protocol.

### Microenvironment-controlled redistribution and redeposition in confined electrochemical architectures

Dissolution redeposition should not be viewed exclusively as a local catalyst-on-catalyst process. In practical electrochemical devices, once dissolved, metal species can leave the parent catalyst and be transported through the electrolyte, ionomer, membrane, or porous electrode architecture before precipitating elsewhere.^205^ The key question is therefore not only whether redeposition occurs but where it occurs, over what distance, and how the local device environment biases that trajectory. Ion concentration gradients, local pH, gas crossover, ionomer interactions, and geometrical confinement all influence whether dissolved species remain near their parent site or migrate over longer distances before re-emerging. In this sense, dissolution and redeposition are governed not only by the metal itself but also by the coupled chemical and transport environment surrounding it.

In PEM systems, Pt provides the clearest classical example. Dissolved Pt formed at the cathode does not necessarily return to the original Pt particles but can migrate into the membrane and precipitate there as the well-known Pt band. Its position is governed by the interplay between Pt-ion transport and locally reducing conditions created by crossover gases, especially H_2_ from the anode. This makes Pt-band formation a particularly clear demonstration that redeposition can be device scale and spatially decoupled from the original dissolution event.^206^^,^^207^^,^^208^

**Figure 10:**
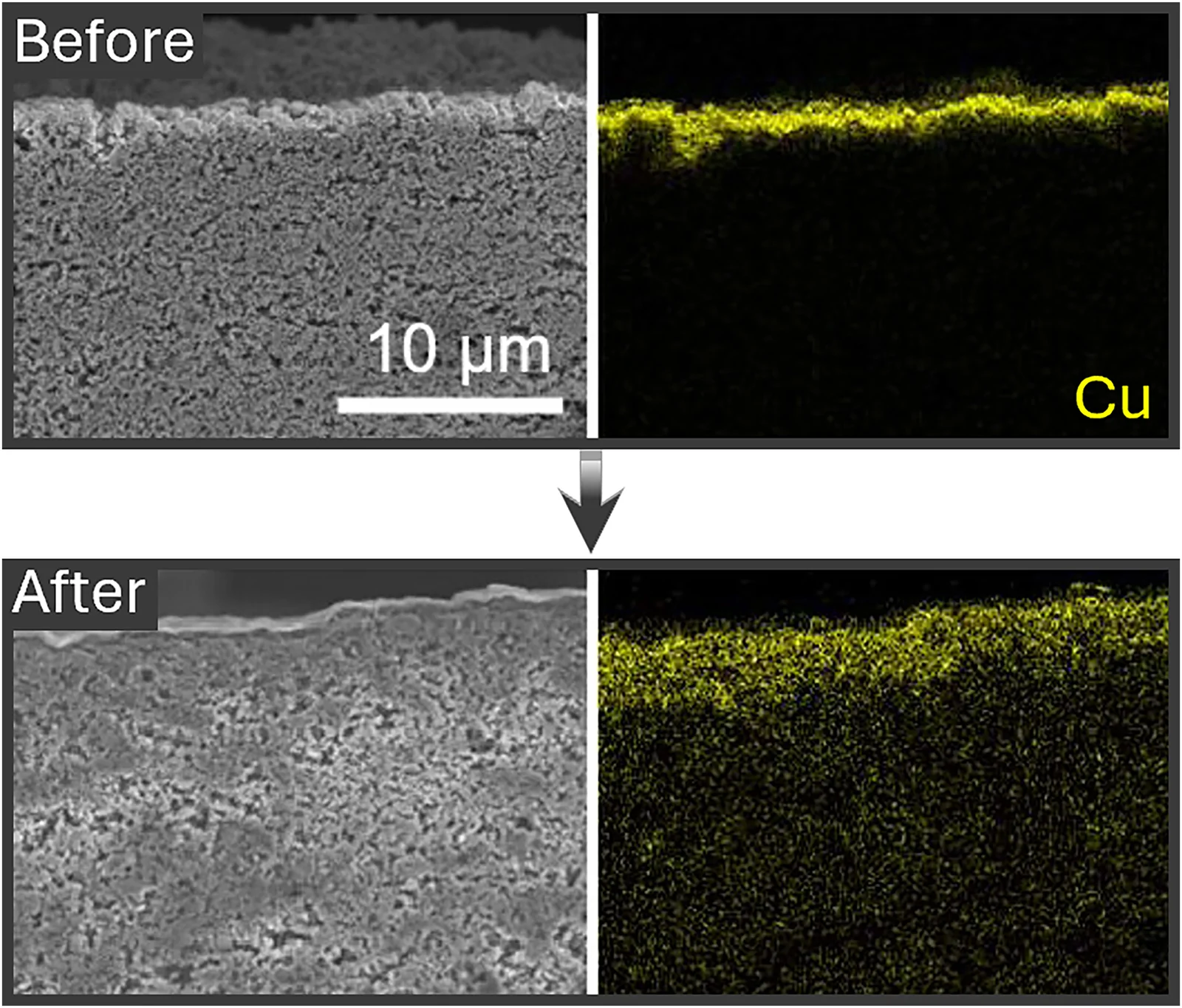
Migration and redeposition of Cu within GDE during CO_2_RR Adapted from Takamatsu et al.,^209^ licensed under CC BY 4.0.

A similar principle has now been demonstrated for Cu gas-diffusion electrodes (GDEs) during CO_2_RR.^209^ In that case, Cu does not remain confined to the initial catalyst sites but redistributes within the GDE during operation (Figure 10). EDS analysis of the GDE cross-sections before and after experiments revealed substantial Cu relocation from the catalyst layer into the gas-diffusion layer. Thus, even under strongly reducing CO_2_RR conditions, catalyst evolution can involve migration through the porous electrode and reprecipitation at new locations.

These examples also highlight why local factors deserve more explicit attention in practical devices. Ion concentration gradients, gas crossover, ionomer- or membrane-mediated transport, and confined geometries all determine whether dissolved species remain near their parent site or migrate over longer distances before redepositing. Recent work on realistic GDE degradation further shows that removing the membrane and exposing the catalyst layer directly to bulk liquid electrolyte amplifies dissolution-migration-redeposition dynamics, whereas membrane-confined architectures restrict transport pathways and produce more localized, representative restructuring.^210^ In other words, dissolution is governed not only by the metal itself but also by the coupled chemical and transport environment surrounding it. For Cu-based CO_2_RR GDEs, nonuniform potential gradients were shown to drive directional in-plane migration of dissolved Cu species, causing porosity changes, catalyst accumulation, and local blockage of CO_2_ transport; notably, these effects were mitigated by increasing local alkalinity, improving potential homogeneity, or confining Cu species with ionomer and overlayer designs.^178^ Likewise, for Ir anodes in CO/CO_2_ electrolysis, crossover of cathodic liquid products such as ethanol and acetaldehyde to the anode side strongly accelerated Ir dissolution by modifying the local reaction environment and suppressing passivating oxide formation, while dissolved Ir was found to accumulate in the membrane and redeposit on the cathode.^107^ These studies show that dissolution redeposition in real devices is not solely an intrinsic property of the metal catalyst but a consequence of the coupled microenvironment created by membranes, ionomers, crossover species, and geometrical confinement, which together determine whether dissolved species redeposit locally or are redistributed across the device.

Recognizing this broader redistribution behavior is important not only for diagnosing degradation but also for controlling it. Once the relevant transport and reduction pathways are understood, they may be mitigated or even steered. For Pt in PEM devices, for example, the reported dependence of Pt-band position on fluidic conditions and potential history suggests that the precipitation zone could, in principle, be shifted by changing gas flow, stoichiometry, or crossover conditions.^207^^,^^211^^,^^212^ More generally, membrane properties, ionomer distribution, local wetting, and electrode confinement should be viewed as design variables that can either suppress harmful long-range migration or redirect restructuring into less damaging pathways.

## Conclusions and outlook

Across essentially all metal and metal-derived electrocatalysts, the interface is not static on relevant operating timescales: atoms can dissolve, migrate, and redeposit, continuously reshaping morphology, composition, and the distribution of active sites. This dissolution-transport-redeposition loop reconciles observations that otherwise appear contradictory, e.g., “stable” Pourbaix windows coexisting with measurable metal fluxes and pronounced restructuring, and it explains why performance and “stability” are often history dependent rather than intrinsic material constants. In this framing, dissolution is rarely a steady background process; it is frequently transient, triggered by specific electrochemical excursions (oxide formation/reduction, start-stop events, and potential cycling) and amplified by local interfacial conditions (pH gradients, complexing ligands/intermediates, and impurities). Importantly, net mass loss is not the sole—or even best—descriptor of durability: redeposition and redistribution can mask overall metal loss while still driving major structural drift, particle growth, porosity evolution, and device-level failure modes such as membrane contamination and catalyst-layer inhomogeneity.

A key message of this perspective is a shift in the stability question. Rather than asking whether a catalyst “maintains its structure,” a more actionable question is what steady state of atom exchange is established under a given operating protocol, and does it preserve (or improve) function? This naturally leads to dynamic stability, where durability can arise not from suppressing atomic mobility entirely (often unrealistic for metals under bias) but from balancing dissolution and redeposition or steering restructuring into benign/self-regenerating trajectories. This perspective also connects electrocatalysis back to corrosion science and electrodeposition: the same elementary steps govern degradation, ripening, reactivation, break-in, and even metal recovery, suggesting that stability engineering and circularity can be treated within one unified framework of atom flow.

Looking ahead, progress will depend less on any single “best” characterization method and more on closing the loop experimentally and conceptually: measuring (1) when atoms leave (flux), (2) where they go and reappear (spatial redistribution), and (3) in what chemical form (speciation/oxidation state/adsorbates) under realistic operation. The correlative toolbox emphasized here of online ICP-MS (time-resolved dissolution), IL-EM (spatial continuity), and *operando*/*in situ* spectroscopy (chemical state and intermediates) provides exactly this closure when deployed as an integrated workflow rather than as isolated snapshots. A key outlook challenge is to translate this correlative philosophy from model electrodes to device-relevant architectures, including GDEs, MEAs, and zero-gap electrolyzers, where transport, ionomer chemistry, local water activity, and spatial gradients fundamentally reshape dissolution and redeposition pathways.

In summary, treating dissolution-transport-redeposition as a universal and controllable motif reframes electrocatalyst stability from “prevent change” to “control atom exchange.” This reframing is not merely semantic: it aligns degradation science with realistic operating protocols, explains why static structure-activity relationships often fail, and opens a pathway to durability strategies that are compatible with intermittent operation and resource constraints. The most impactful next advances will likely come from workflows that unify flux, structure, and chemistry under device-relevant conditions, turning dynamic restructuring from an interpretational ambiguity into a design variable for long-lived, circular electrocatalytic technologies.

## Acknowledgments

The authors would like to acknowledge the Slovenian Research and Innovation Agency (ARIS) through programs P2-0132, P2-0421, P2-0393, I0-0003, and I0-0006 and projects GC-0004, N2-0155, N2-0248, N2-0337, N2-0425, J1-4401, J7-4638, and J2-60043; the 10.13039/501100000781European Research Council (ERC) Starting Grant 123STABLE (grant agreement ID: 852208); and the North Atlantic Treaty Organization Science for Peace and Security (NATO SPS) program (grant G6230).

## Author contributions

Conceptualization, B.T., M. Smiljanić, and N.H.; writing – original draft, all authors; writing – review & editing, B.T., M. Smiljanić, and N.H.; and funding acquisition, N.H.

## Declaration of interests

The authors declare that they have no competing financial interests or personal relationships that could have appeared to influence the work reported in this paper. The research was conducted independently of the funding bodies, which had no role in the study design, data analysis, interpretation, or decision to publish.
